# S100A9 modulates USP7-mediated stabilization of NCOA4 to promote ferroptosis in sepsis-associated acute lung injury

**DOI:** 10.1016/j.redox.2026.104271

**Published:** 2026-06-21

**Authors:** Yi Wei, Angran Gu, Bailun Wang, Yizheng Yang, Chang Sun, Yi Zhang, Runmeng Liu, Yichen Wang, Changping Gu, Yuelan Wang

**Affiliations:** a Department of Anesthesiology, Shandong Provincial Hospital Affiliated to Shandong First Medical University, Jinan, Shandong, 250021, China; b Shandong First Medical University, Jinan, Shandong, 250021, China

**Keywords:** Sepsis-associated acute lung injury, Ferroptosis, Ferritinophagy, S100A9, USP7, NCOA4

## Abstract

Sepsis-associated acute lung injury (SALI) is driven by dysregulated macrophage activation; however, mechanisms linking innate immune signaling to cell death remain elusive. Emerging evidence implicates ferroptosis, fueled by NCOA4-mediated ferritinophagy, as a critical executioner of macrophage death. Yet, post-translational mechanisms dictating NCOA4 stability and preventing its premature degradation during sepsis are poorly understood. Specifically, how damage-associated molecular patterns (DAMPs) like S100A9 sustain this pro-ferroptotic flux remains unknown. Here, we identify an S100A9–USP7–NCOA4 axis linking DAMP-mediated inflammation to ferritinophagy-dependent ferroptosis in alveolar macrophages. Sepsis-induced S100A9 acts as an intracellular scaffold, recruiting the deubiquitinase USP7 to NCOA4. USP7 cleaves K63-linked polyubiquitin chains at NCOA4 residues K42 and K181, preventing autophagic degradation and sustaining ferritin catabolism, iron release, and lipid peroxidation. Molecular docking reveals S100A9 optimally positions USP7 near NCOA4 K181 for site-specific deubiquitination. Crucially, S100A9 ablation or pharmacological USP7 inhibition with P5091 disrupts this axis, suppressing ferroptosis and alleviating lung injury in a murine cecal ligation and puncture (CLP) model. Clinically, USP7 and NCOA4 are positively co-expressed in sepsis patients, correlating with reduced 28-day survival. Collectively, our findings establish ferritinophagy as a bridge between innate immunity and ferroptosis in SALI, highlighting USP7 as a mechanistically defined, actionable therapeutic target.

## Highlights

• S100A9 acts as an intracellular scaffold linking DAMP signaling to ferroptosis in SALI.
• USP7 removes K63-linked ubiquitin from NCOA4 at K42/K181, preventing autophagic degradation.
• S100A9–USP7–NCOA4 axis sustains ferritinophagy, iron release, and lipid peroxidation.
• USP7 inhibition with P5091 suppresses ferroptosis and alleviates lung injury in CLP mice.
• USP7 and NCOA4 co-expression correlates with reduced 28-day survival in sepsis patients.

## 1 Introduction

Sepsis is a life-threatening organ dysfunction caused by a dysregulated host response to infection [1]. One of its most severe complications is sepsis-associated acute lung injury (SALI) or acute respiratory distress syndrome (ARDS) [2], characterized by acute respiratory failure resulting from uncontrolled inflammation, loss of endothelial barrier integrity, and severe tissue edema [3]. Despite significant efforts in developing targeted therapies, most of these specific treatments have failed to reduce mortality in clinical trials, highlighting the urgent need to identify and exploit novel molecular drivers in the pathogenesis of SALI for therapeutic purposes. A granular molecular understanding of the regulated cell death pathways operative in macrophages during sepsis is therefore critical for identifying novel therapeutic targets [4].

Ferroptosis is an iron-dependent, non-apoptotic form of regulated cell death driven by the unrestricted peroxidation of polyunsaturated fatty acid-containing phospholipids. Mechanistically, ferroptosis is an iron-driven cell death caused by the accumulation of reactive oxygen species (ROS) and unconstrained lipid peroxidation [5]. This process is counterbalanced by glutathione peroxidase 4 (GPX4), the principal barrier against membrane damage, and exacerbated by acyl-CoA synthetase long chain family member 4 (ACSL4), which enriches cellular membranes with oxidation-prone arachidonic acid [6]. Emerging evidence implicates ferroptosis as a critical driver of organ damage in sepsis; elevated markers of lipid peroxidation and iron dysregulation have been documented in the lungs, kidneys, and livers of both septic patients and experimental models [7,8]. Yet the upstream molecular signals that initiate ferroptosis in alveolar macrophages during sepsis remain incompletely defined.

A central mechanism coupling iron homeostasis to ferroptosis susceptibility is ferritinophagy—the selective autophagic degradation of ferritin, the primary intracellular iron storage complex [9]. Nuclear receptor coactivator 4 (NCOA4) functions as the cargo receptor for ferritinophagy, binding ferritin heavy chain 1 (FTH1) and delivering ferritin complexes to autophagosomes for lysosomal degradation. The resultant liberation of labile Fe^2+^ from ferritin provides the iron pool that fuels lipid peroxidation and ferroptotic death [10,11]. NCOA4 protein abundance is tightly regulated by ubiquitin-mediated proteasomal and autophagic pathways; under iron-replete conditions, NCOA4 itself is targeted for degradation to limit ferritinophagy [12,13]. Critically, the deubiquitinase landscape governing NCOA4 stability in the context of inflammatory injury has not been systematically explored.

S100A9 (also known as MRP14 or calgranulin-B) is a calcium- and zinc-binding protein of the S100 family that is abundantly expressed in and released from myeloid cells, particularly alveolar macrophages and neutrophils, under inflammatory conditions [[14], [15], [16]]. Clinical and experimental studies have shown that S100A9 expression is significantly upregulated during systemic infections, and its circulating levels are positively correlated with the severity of SALI, cytokine storm, and poor prognosis [17,18]. During sepsis, S100A9 is markedly upregulated and functions as a damage-associated molecular pattern (DAMP), activating Toll-like receptor 4 (TLR4) and receptor for advanced glycation end products (RAGE) signaling to amplify downstream inflammatory cascades, driving macrophage hyperactivation, oxidative stress, and the exacerbation of pathological lung tissue damage [19]. Beyond its extracellular cytokine-like functions, emerging evidence suggests intracellular roles for S100A9 as a molecular scaffold that modulates protein–protein interactions and post-translational modifications [[20], [21], [22]]. However, whether intracellular S100A9 directly regulates ferritinophagy and ferroptosis through modification of cargo receptor stability has not been investigated.

Ubiquitin-specific protease 7 (USP7, also known as HAUSP) is one of the best-characterized deubiquitinases in mammalian cells, regulating the stability of diverse substrates including p53, MDM2, PTEN, and FOXO proteins [23]. Recent studies have shown that the deubiquitinase USP7 plays a key regulatory role in the pathophysiological processes of sepsis-induced organ injury, particularly acute lung injury. In the sepsis microenvironment, abnormally upregulated USP7 stabilizes key proteins such as PDK1 and CXCL3 through deubiquitination, leading to excessive activation and pro-inflammatory polarization of vascular endothelial cells, thereby significantly amplifying the pulmonary inflammatory cascade [24,25]. In addition to classical inflammatory pathways, USP7 is also deeply involved in the complex regulatory network of the ferroptosis pathway. Studies have shown that USP7 can both inhibit YAP transcriptional activity by stabilizing LATS1, thereby indirectly downregulating the expression of core anti-ferroptotic factors GPX4 and SLC7A11, and, under specific conditions, directly maintain the protein stability of GPX4 within macrophages [26,27]. However, the precise substrates and molecular mechanisms by which USP7 exerts these functions in SALI remain unclear. Notably, USP7 can remove K63-linked polyubiquitin chains from substrates, a modification closely associated with the recognition of autophagic cargo and selective autophagic degradation [28,29]. Considering the expanding spectrum of USP7 substrates, it may regulate ferritin autophagy by deubiquitinating NCOA4.

Here, we demonstrate that S100A9 is critical for driving NCOA4-dependent ferritin autophagy and ferroptosis in alveolar macrophages during sepsis. Mechanistically, S100A9 acts as a key molecular scaffold, recruiting the deubiquitinase USP7 to NCOA4, thereby promoting the removal of K63-linked polyubiquitin chains from lysine residues K42 and K181 on NCOA4. This, in turn, inhibits the autophagic degradation of the ferritinophagy receptor, and promotes ferritin catabolism, iron release, and lipid peroxidation. Pharmacological inhibition of USP7 with P5091 disrupts this axis, reduces NCOA4 abundance and ferritinophagy, and significantly alleviates SALI in vivo. Our findings identify a previously unrecognized S100A9–USP7–NCOA4 regulatory axis that links innate immune activation to macrophage ferroptosis and establish USP7 as a druggable target for therapeutic interventions against SALI.

## 2 Results

### 2.1 S100A9 promotes ferritinophagy and ferroptosis in acute lung injury

**Fig. 1:**
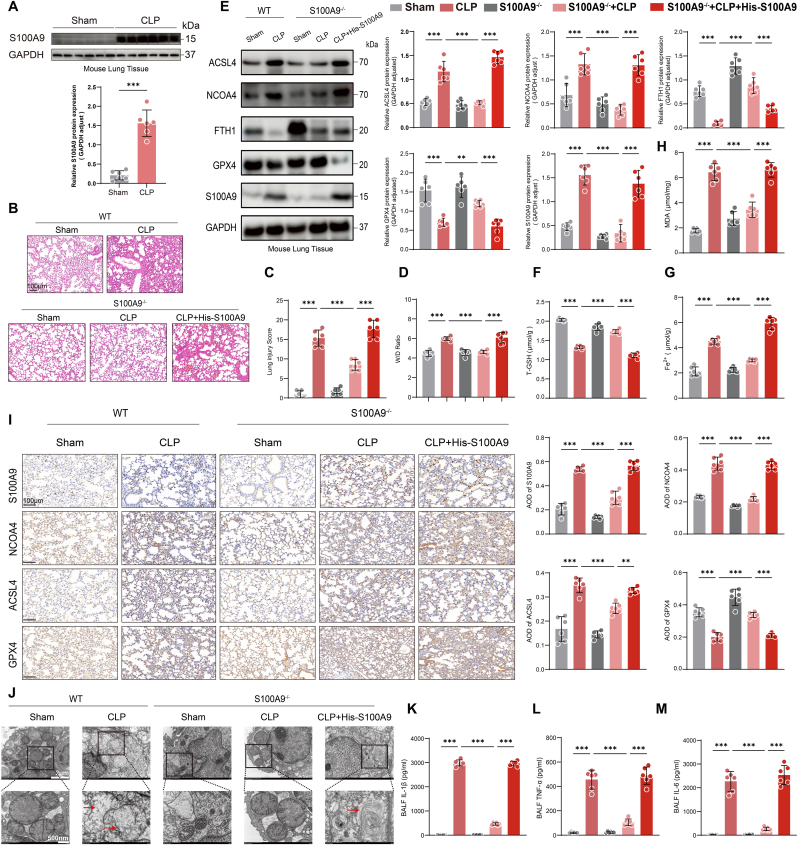
**S100A9 deficiency attenuates sepsis-induced ferroptosis and lung injury in vivo. (A)** Western blot analysis and quantification of S100A9 protein expression in lung tissues from Sham and CLP-induced WT mice (n = 6). **(B)** Representative H&E-stained lung sections from WT (Sham and CLP; upper panels) and S100A9^−/−^ (Sham, CLP, and CLP + His-S100A9; lower panels) mice. Scale bar, 100 μm. **(C)** Lung injury scores quantified from histological sections in (B) (n = 6). **(D)** Lung W/D weight ratio as an index of pulmonary edema (n = 6). **(E)** Immunoblot analysis of ACSL4, NCOA4, FTH1, GPX4, S100A9 in lung tissue from WT (Sham and CLP) and S100A9^−/−^ (Sham, CLP, and CLP + His-S100A9) mice (n = 6). **(F)** T-GSH content in lung tissue (μmol/g) across the indicated groups (n = 6). **(G)** Labile Fe^2+^ levels (μmol/g) in lung tissue (n = 6). **(H)** MDA content (μmol/mg) in lung tissue (n = 6). **(I)** Representative IHC images of S100A9, NCOA4, ACSL4, and GPX4 in lung sections from all groups. Scale bar, 100 μm. Quantifications of average optical density (AOD) are shown in the adjacent bar graphs (n = 6). **(J)** Representative TEM images of lung tissues from all groups. Lower panels show high-magnification insets of the boxed regions; red arrows indicate mitochondria exhibiting ferroptotic morphological changes (condensed, electron-dense, with disrupted cristae). Scale bar, 2 μm/500 nm. **(K** to **M)** BALF concentrations of IL-1β (K), TNF-α (L), and IL-6 (M) measured by ELISA (n = 6). Data are presented as mean ± SD. Statistical significance was determined using an unpaired two-tailed *t*-test for panel (A), and an ordinary one-way ANOVA followed by Tukey's multiple comparisons test for multi-group comparisons in panels (C to I, and K to M). ∗∗*P* < 0.01, ∗∗∗*P* < 0.001.

To investigate the in vivo role of S100A9 in sepsis-associated ferroptosis, we first confirmed that S100A9 protein was significantly upregulated in the lungs of wild-type (WT) mice subjected to cecal ligation and puncture (CLP) compared with sham-operated controls (Fig. 1A). We then subjected S100A9-knockout mice (S100A9^−/−^) and WT C57BL/6J mice to CLP, and assessed lung injury, ferroptosis markers, and inflammatory responses. Histological examination by H&E staining revealed that CLP induced prominent alveolar wall thickening, interstitial edema, and leukocyte infiltration in WT mice; these pathological features were markedly attenuated in S100A9^−/−^ mice (Fig. 1B and C). Consistent with reduced tissue injury, the pulmonary wet-to-dry (W/D) weight ratio was significantly lower in CLP-challenged S100A9^−/−^ mice compared with WT controls (Fig. 1D).

Western blot analysis of lung tissue demonstrated that CLP-induced upregulation of ACSL4 and NCOA4, and downregulation of FTH1 and GPX4, were all substantially reversed in S100A9^−/−^ mice (Fig. 1E). Biochemical markers of ferroptosis, including MDA content, labile Fe^2+^ levels, and total glutathione (T-GSH), further confirmed that S100A9 deficiency significantly attenuated CLP-induced lipid peroxidation and iron dysregulation (Fig. 1F–H). Immunohistochemical (IHC) staining corroborated these findings at the tissue level: the CLP-induced increases in S100A9, NCOA4, and ACSL4 abundance, and the decrease in GPX4, were all reversed in S100A9^−/−^ lungs (Fig. 1I). Transmission electron microscopy (TEM) of lung tissue revealed that CLP induced characteristic ferroptotic mitochondrial changes, including mitochondrial shrinkage, increased electron density, and cristae disruption, which were largely absent in S100A9^−/−^ mice (Fig. 1J). Furthermore, BALF levels of the proinflammatory cytokines IL-1β, TNF-α, and IL-6 were significantly reduced in CLP-challenged S100A9^−/−^ mice relative to WT counterparts (Fig. 1K–M).

To confirm that these protective effects were caused by the absence of S100A9, we conducted a protein supplementation experiment in which we injected the recombinant His-S100A9 protein into CLP-challenged mice. His-S100A9 supplementation restored ferroptosis markers (elevated ACSL4 and NCOA4; reduced FTH1, GPX4, and T-GSH), exacerbated lung histopathology, increased the W/D ratio, elevated MDA and Fe^2+^ levels, and augmented BALF cytokine concentrations to levels comparable to those observed in CLP-challenged WT mice (Fig. 1B–M). Collectively, these data demonstrate that S100A9 plays a critical role in driving ferroptosis and inflammatory lung injury in the CLP model of sepsis.

### 2.2 S100A9 knockdown alleviates LPS-induced ferroptosis in vitro

**Fig. 2:**
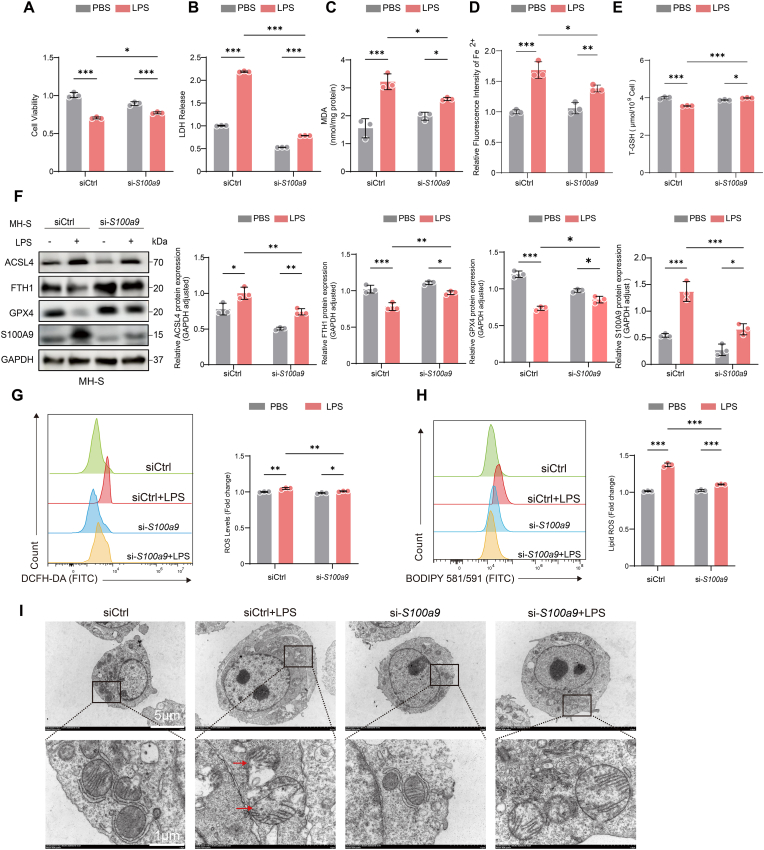
***S100a9* knockdown attenuates LPS-induced ferroptosis in macrophages. (A** and **B)** Cell viability (A) and LDH release (B) were measured in MH-S cells across the indicated treatment groups. **(C** to **E)** Quantification of intracellular MDA levels (C), relative fluorescence intensity of ferrous iron (D), and T-GSH levels (E) in MH-S cells. **(F)** Representative immunoblots of ACSL4, FTH1, GPX4, and S100A9 protein expression in siCtrl and si-*S100a9* MH-S cells treated with PBS or LPS. **(G)** Representative flow cytometry histograms of DCFH-DA (total ROS) fluorescence in MH-S cells under the indicated conditions. **(H)** Representative flow cytometry histograms of BODIPY 581/591 (lipid ROS) staining. **(I)** Representative TEM images of MH-S cells under the indicated conditions. Lower panels show high-magnification insets; red arrows indicate mitochondria with characteristic ferroptotic morphology (shrunken, electron-dense, with cristae loss). Scale bar, 5 μm/1 μm. Data are presented as mean ± SD from at least three independent experiments. Statistical significance was assessed by one-way ANOVA with Tukey's post hoc test. ∗*P* < 0.05, ∗∗*P* < 0.01, ∗∗∗*P* < 0.001.

To further validate the role of S100A9 in mediating cellular damage, we utilized an in vitro model by transfecting MH-S alveolar macrophages with small interfering RNA against *S100a9* (si-*S100a9*) prior to lipopolysaccharide (LPS) stimulation. LPS treatment significantly reduced cell viability and provoked substantial lactate dehydrogenase (LDH) release, whereas silencing *S100a9* effectively mitigated these cytotoxic effects (Fig. 2A and B). We subsequently examined classical biochemical markers of lipid peroxidation and ferroptosis. *S100a9* knockdown reversed the LPS-induced intracellular accumulation of MDA and ferrous iron (Fig. 2C and D), while successfully restoring T-GSH levels (Fig. 2E). Consistent with these biochemical alterations, Western blot analysis confirmed the successful knockdown of S100A9 and revealed that si-*S100a9* counteracted the LPS-triggered upregulation of ACSL4 and the concurrent downregulation of both FTH1 and GPX4 protein expression (Fig. 2F). Flow cytometric measurement of cellular ROS (DCFH-DA) and lipid peroxidation (BODIPY 581/591) confirmed that S100a9 silencing significantly suppressed LPS-induced increases in both total ROS and lipid ROS (Fig. 2G and H). TEM further revealed that LPS treatment produced characteristic ferroptotic mitochondrial morphology, including mitochondrial atrophy, increased membrane density, and cristae fragmentation; these features were significantly alleviated following *S100a9* knockdown (Fig. 2I). To delineate the underlying regulatory mechanism, we evaluated the transcriptional profiles of these markers. S100a9 knockdown efficiently rescued the LPS-induced transcriptional shifts in Acsl4, Fth1, and Gpx4 (Fig. S1A and C-E). Collectively, these findings validate that S100A9 plays a key role in regulating ferroptosis in alveolar macrophages.

To further determine whether this phenotype was preserved in primary macrophages, we performed parallel validation experiments using primary BMDMs isolated from WT and S100a9-deficient mice. Consistent with the observations in MH-S cells, LPS stimulation increased ACSL4 expression, intracellular Fe^2+^ accumulation, MDA production, LDH release, total ROS, and lipid ROS levels, while reducing FTH1, GPX4, T-GSH, and cell viability in WT BMDMs. These ferroptosis-associated changes were markedly attenuated in S100a9-deficient BMDMs (Fig. S2A–H). These results support that S100A9 deficiency protects primary macrophages from LPS-induced ferroptotic injury.

### 2.3 S100A9 drives ferroptosis through NCOA4-mediated ferritinophagy

**Fig. 3:**
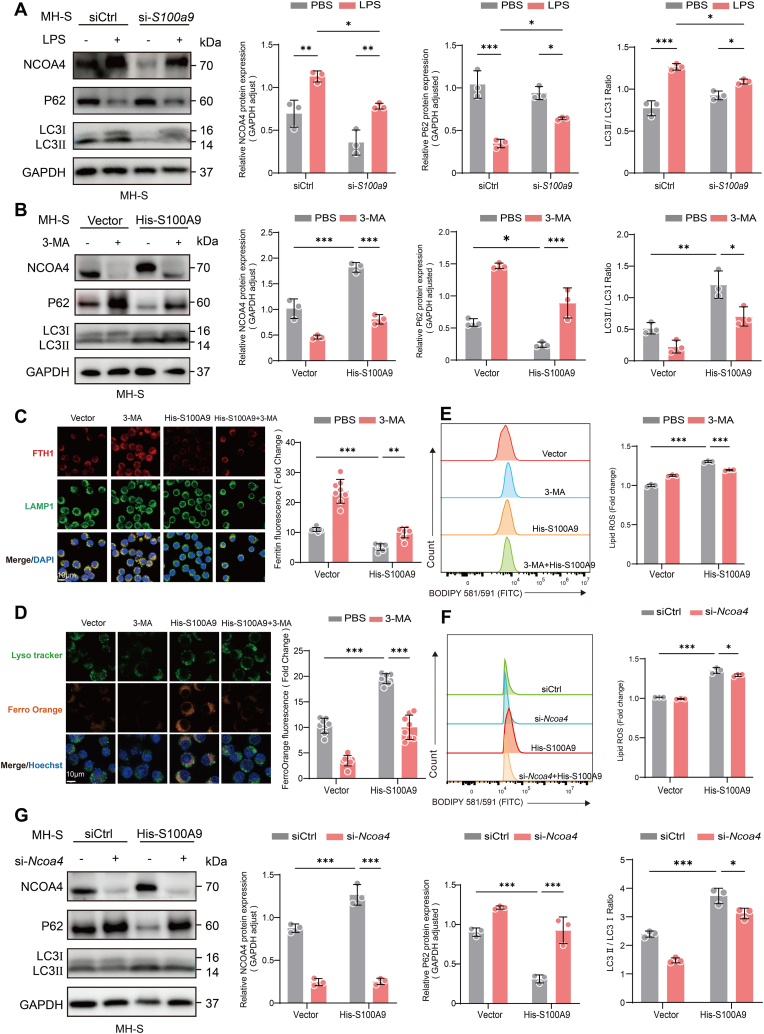
**S100A9 promotes macrophage ferroptosis via NCOA4-dependent ferritinophagy. (A)** Western blot analysis and corresponding quantification of NCOA4, p62, and LC3 in LPS-treated MH-S cells transfected with siCtrl or si-*S100a9*. **(B)** Western blot and quantification of NCOA4, p62, and LC3 in MH-S cells treated with recombinant His-S100A9 and/or the autophagy inhibitor 3-MA. **(C)** Immunofluorescence co-staining of FTH1 (red) and LAMP1 (green) in MH-S cells under the indicated conditions (Vector, 3-MA, His-S100A9, His-S100A9+3-MA). Nuclei stained with DAPI (blue). Scale bar, 10 μm. Quantification of ferritin fluorescence intensity per cell is shown on the right (n = 8). **(D)** Fluorescence co-staining of LysoTracker (green) and FerroOrange (orange) in MH-S cells under the indicated conditions. Nuclei stained with Hoechst (blue). Scale bar, 10 μm. Quantification of FerroOrange fluorescence intensity is shown on the right (n = 8). **(E)** Representative flow cytometry histograms of BODIPY 581/591 (lipid ROS) staining in MH-S cells transfected with vector or His-S100A9 and treated with PBS or 3-MA, with quantification of lipid ROS fold change. **(F)** Representative flow cytometry histograms of BODIPY 581/591 in MH-S cells with si-*Ncoa4* and/or His-S100A9 overexpression, with quantification of lipid ROS fold change. **(G)** Western blot and relative quantification of NCOA4, p62, and LC3 in MH-S cells overexpressing vector or His-S100A9 with siCtrl or si-*Ncoa4* treatment. Data are presented as mean ± SD from at least three independent experiments. Statistical significance was assessed by two-way ANOVA with Tukey's post hoc test. ∗*P* < 0.05, ∗∗*P* < 0.01, ∗∗∗*P* < 0.001.

Having established the essential role of S100A9 in ferroptosis, we next investigated the underlying mechanisms governing autophagic flux and ferritinophagy. In LPS-stimulated MH-S cells, *S100a9* knockdown reduced NCOA4 protein levels, reduced autophagy flux—as evidenced by elevated p62 levels and a decreased LC3-II/LC3-I ratio—and attenuated ferroptosis markers (Fig. 3A). Conversely, His-S100A9 overexpression increased NCOA4, promoted autophagy, and increased ferritinophagy markers; 3-methyladenine (3-MA) treatment abrogated these effects (Fig. 3B). Notably, although S100A9 significantly regulated the accumulation of the NCOA4 protein, the mRNA levels of the ferritinophagy receptor Ncoa4 remained unchanged across all groups (Fig. S1B). This discrepancy suggests that S100A9 regulates NCOA4 abundance exclusively at the post-translational level. These results indicate that S100A9 promotes NCOA4-dependent ferritinophagy via the autophagy-lysosomal pathway.

To assess whether S100A9-driven ferritinophagy ultimately leads to ferroptosis, we examined a series of ferroptosis markers in His-S100A9-overexpressing MH-S cells. S100A9 overexpression significantly increased LDH release, MDA content, and labile Fe^2+^ levels, while reducing T-GSH, indicative of robust ferroptosis; all of these effects were substantially reversed by 3-MA-mediated autophagy blockade (Fig. S3A–D). These data confirm that the pro-ferroptotic activity of S100A9 is dependent on intact autophagic flux.

To determine whether these effects required NCOA4, we performed *Ncoa4* knockdown in the context of His-S100A9 overexpression. *Ncoa4* silencing significantly rescued LDH release, MDA accumulation, Fe^2+^ elevation, and T-GSH depletion induced by S100A9 overexpression (Fig. S3E–H), establishing NCOA4 as an essential downstream effector of S100A9-mediated ferroptosis.

To further dissect the ferritinophagy mechanism, we performed immunofluorescence co-staining of FTH1 and the lysosomal marker LAMP1. LPS stimulation markedly increased the colocalization of FTH1 with LAMP1, indicating enhanced lysosomal ferritin trafficking; *S100a9* knockdown significantly attenuated this response (Fig. S3I). Consistently, co-staining with LysoTracker and FerroOrange demonstrated that LPS-induced lysosomal iron accumulation was substantially reduced by *S100a9* silencing (Fig. S3J). His-S100A9 overexpression markedly increased FTH1–LAMP1 co-localization and lysosomal iron accumulation, both of which were significantly reversed by *Ncoa4* knockdown (Fig. S3K–L), confirming that S100A9 drives lysosomal ferritin degradation and iron release in a strictly NCOA4-dependent manner.

This conclusion was further corroborated by data from Fig. 3C and D, in which His-S100A9-driven increases in FTH1 lysosomal trafficking and FerroOrange-positive lysosomal iron were abolished by 3-MA treatment. Flow cytometric analysis of lipid ROS (BODIPY 581/591) showed that S100A9 overexpression significantly elevated lipid peroxidation, an effect abolished by 3-MA (Fig. 3E). Critically, *Ncoa4* knockdown rescued lipid ROS accumulation induced by His-S100A9 (Fig. 3F), and concomitantly restored p62 levels and reduced the LC3-II/LC3-I ratio (Fig. 3G), confirming that NCOA4 is indispensable downstream of S100A9 for ferritinophagy-driven ferroptosis. Collectively, these data establish that S100A9 promotes ferritinophagy by stabilizing NCOA4, leading to lysosomal FTH1 degradation, labile iron release, lipid ROS accumulation, and ultimately macrophage ferroptosis.

The regulatory effect of S100A9 on NCOA4-associated ferritinophagy was also validated in primary BMDMs. LPS stimulation increased NCOA4 protein abundance, reduced p62 expression, and increased the LC3-II/LC3-I ratio in WT BMDMs, consistent with activation of autophagy/ferritinophagy. In contrast, S100a9 deficiency blunted LPS-induced NCOA4 accumulation, partially restored p62 expression, and attenuated LC3 conversion (Fig. S4A–D). These findings indicate that S100A9 is required for efficient LPS-induced NCOA4 accumulation and ferritinophagy-associated autophagic activation in primary macrophages.

### 2.4 S100A9 interacts with NCOA4 and attenuates its K63-linked ubiquitination

**Fig. 4:**
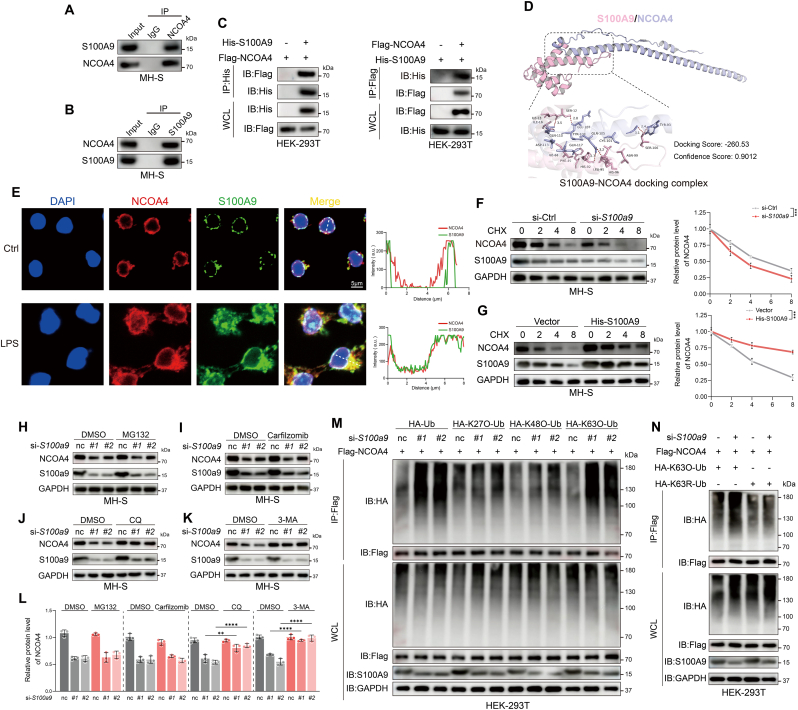
**S100A9 interacts with NCOA4 and attenuates its K63-linked ubiquitination and autophagic degradation. (A to B)** Endogenous Co-IP in MH-S cells. Cell lysates were immunoprecipitated with anti-NCOA4 (A) or anti-S100A9 (B) antibodies and immunoblotted for the indicated proteins. IgG served as negative control. **(C)** Reciprocal Co-IP in HEK-293T cells overexpressing His-S100A9 and/or Flag-NCOA4, immunoprecipitated with anti-His (left) or anti-Flag (right) antibodies. **(D)** Molecular docking model of the S100A9–NCOA4 complex. S100A9 is shown in pink; NCOA4 in purple. Key interacting residues are indicated. Docking score: −260.53; confidence score: 0.9012. **(E)** Representative confocal immunofluorescence images and corresponding fluorescence line-scan profiles showing the colocalization of S100A9 (green) and NCOA4 (red) in MH-S cells treated with or without LPS. Scale bar, 5 μm. **(F** and **G)** Western blot analysis and relative quantification of NCOA4 protein stability in MH-S cells transfected with si-*S100a9* (F) or His-S100A9 (G) and treated with CHX (100 μg/mL) for the indicated times. **(H** to **K)** MH-S cells transfected with si-*S100a9* (#1, #2) or negative control (nc) were treated with the proteasome inhibitors MG132 (10 μM) (H) or carfilzomib (100 nM) (I), or the autophagy inhibitors CQ (50 μM) (J) or 3-MA (10 mM) (K) alongside DMSO vehicle. Representative immunoblots for NCOA4, S100A9, and GAPDH are shown. **(L)** Quantification of relative NCOA4 protein levels across conditions shown in (H to K). **(M)** Ubiquitination assay in HEK-293T cells co-transfected with Flag-NCOA4, si-*S100a9*, and HA-tagged ubiquitin constructs (HA-Ub, HA-K27O-Ub, HA-K48O-Ub, or HA-K63O-Ub). Immunoprecipitation was performed with anti-Flag antibody; immunoblots were probed for HA and Flag. WCL indicates whole cell lysate. **(N)** Ubiquitination assay in HEK-293T cells co-expressing Flag-NCOA4, si-*S100a9*, and either HA-K63O-Ub or HA-K63R-Ub. Data are presented as mean ± SD; Statistical analysis by two-way ANOVA with Tukey's post hoc test. ∗∗*P* < 0.01, ∗∗∗∗*P* < 0.0001; ns, not significant.

To elucidate the molecular mechanism by which S100A9 regulates NCOA4 stability, we first examined whether S100A9 physically interacts with NCOA4. Endogenous Co-IP assays in MH-S cells demonstrated that S100A9 and NCOA4 reciprocally immunoprecipitated each other (Fig. 4A and B). Exogenous Co-IP experiments in HEK-293T cells co-expressing His-S100A9 and Flag-NCOA4 confirmed this bidirectional interaction (Fig. 4C). Then, to further investigate the specific interaction between S100A9 and NCOA4, we utilized HDOCK for protein-protein docking predictions. The computational analysis predicted a stable S100A9–NCOA4 complex with a docking score of −260.53 and a confidence score of 0.9012 (Fig. 4D), which supported our findings. Immunofluorescence revealed substantial colocalization of NCOA4 (red) and S100A9 (green), which was further enhanced following LPS stimulation (Fig. 4E), suggesting that inflammatory signals promote the assembly of this complex.

Given this direct physical interaction, we hypothesized that S100A9 regulates NCOA4 protein stability. Cycloheximide (CHX) chase assays demonstrated that *S100a9* knockdown significantly accelerated the degradation rate of NCOA4 (Fig. 4F), whereas S100A9 overexpression substantially prolonged its half-life (Fig. 4G). To identify the pathways involved in NCOA4 degradation following *S100a9* silencing, we treated cells with the proteasome inhibitors MG132 and carfilzomib, or the autophagy inhibitors chloroquine (CQ) and 3-MA. Notably, only CQ and 3-MA restored NCOA4 protein levels in *S100a9*-knockdown cells, whereas proteasome inhibitors had no effect, indicating that NCOA4 is degraded via the autophagy-lysosomal pathway in the absence of S100A9 (Fig. 4H–L).

To characterize the specific ubiquitination linkage governing NCOA4 autophagic targeting, we performed ubiquitination assays using HA-tagged ubiquitin constructs with lysine-specific mutations. *S100a9* knockdown preferentially enhanced K63-linked (rather than K27- or K48-linked) polyubiquitination of NCOA4 (Fig. 4M), indicating that S100A9 specifically attenuates K63-linked ubiquitination of NCOA4. Consistent with this, expression of K63O-Ub (all lysines mutated except K63) robustly promoted NCOA4 ubiquitination, whereas K63R-Ub (K63 mutated to arginine) abolished this modification (Fig. 4N). Collectively, these results demonstrate that S100A9 stabilizes NCOA4 by attenuating its K63-linked polyubiquitination and subsequent autophagic degradation.

### 2.5 USP7 deubiquitinates and stabilizes NCOA4

**Fig. 5:**
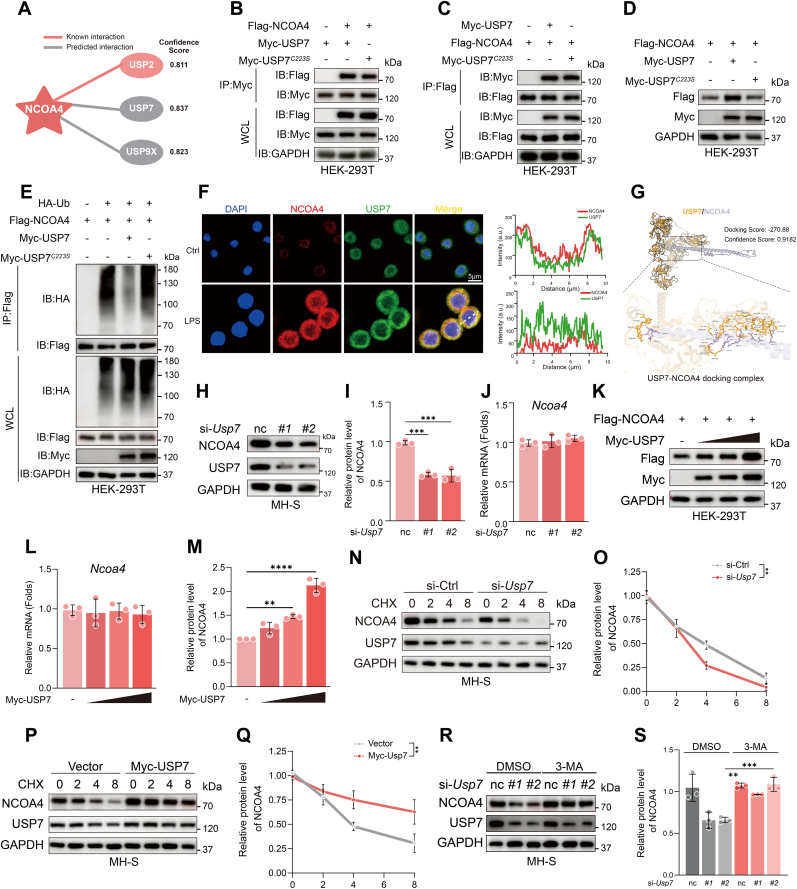
**USP7 deubiquitinates and stabilizes NCOA4 in a catalytic activity-dependent manner. (A)** The NCOA4 deubiquitinase-substrate interaction network was predicted using the UbiBrowser database. This network shows predicted interactions with USP2 (confidence score: 0.811), USP7 (0.837), and USP9X (0.823). Pink lines indicate known interactions; gray lines indicate predicted interactions. **(B** and **C)** HEK-293T cells transfected with Flag-NCOA4 and either Myc-USP7 or Myc-USP7^C223S^ were immunoprecipitated with anti-Myc (B) or anti-Flag (C) antibodies, followed by immunoblotting. **(D)** Immunoblot analysis of NCOA4 protein levels in HEK-293T cells co-expressing Flag-NCOA4 with Myc-USP7 or Myc-USP7^C223S^. **(E)** Ubiquitination assay in HEK-293T cells co-transfected with HA-Ub, Flag-NCOA4, and Myc-USP7 or Myc-USP7^C223S^. Immunoprecipitation with anti-Flag. **(F)** Representative confocal images and fluorescence line-scan profiles showing colocalization of NCOA4 (red) and USP7 (green) in MH-S cells with or without LPS treatment. Scale bar, 5 μm. **(G)** Molecular docking model of the USP7–NCOA4 complex. USP7 is shown in gold; NCOA4 in purple. Docking score: −270.88; confidence score: 0.9182. **(H** and **I)** Western blot (H) and relative quantification (I) of NCOA4 and USP7 in MH-S cells transfected with si-Ctrl or si-*Usp7* (#1, #2). **(J)** RT-qPCR analysis of *Ncoa4* mRNA in MH-S cells transfected with si-Ctrl or si-*Usp7* (#1, #2). **(K** and **M)** Western blot (K) and quantification (M) of NCOA4 protein levels in MH-S cells transfected with increasing doses of Myc-USP7. **(L)** Relative *Ncoa4* mRNA levels in USP7-overexpressing MH-S cells. **(N** and **O)** CHX (100 μg/mL) chase assay (N) and corresponding quantification of relative NCOA4 protein levels (O) in MH-S cells transfected with nc or si-*Usp7*. **(P** and **Q)** CHX (100 μg/mL) chase assay (P) and corresponding quantification of relative NCOA4 protein levels (Q) in MH-S cells transfected with empty vector or Myc-USP7. **(R** and **S)** Immunoblot analysis (R) and corresponding quantification (S) of NCOA4 and USP7 in MH-S cells transfected with nc or si-*Usp7* (#1, #2), followed by treatment with DMSO or 3-MA (10 mM). Data are presented as mean ± SD from at least three independent experiments. Statistical significance was assessed by two-way ANOVA with Tukey's post hoc test. ∗*P* < 0.05, ∗∗*P* < 0.01, ∗∗∗*P* < 0.001, ∗∗∗∗*P* < 0.0001; ns, not significant.

To identify the deubiquitinase(s) responsible for NCOA4 stabilization, we queried the UbiBrowser database, which predicted USP7 (confidence score: 0.837), USP9X (0.823), and USP2 (0.811) as candidate interactors of NCOA4 (Fig. 5A). Given that USP7 exhibited the highest confidence score and has been increasingly reported to play crucial regulatory roles in both sepsis-induced acute lung injury and ferroptosis [[24], [25], [26], [27]], we selected it as the primary candidate for further investigation. Co-IP experiments in HEK-293T cells confirmed that WT Myc-USP7 and the catalytically inactive mutant Myc-USP7^C223S^ [30,31], interacted with Flag-NCOA4, demonstrating that USP7 binds to NCOA4 independently of its catalytic activity (Fig. 5B and C). In contrast to WT USP7, overexpression of USP7^C223S^ failed to stabilize NCOA4 protein levels (Fig. 5D). Ubiquitination assays revealed that WT USP7 significantly reduced Flag-NCOA4 ubiquitination levels, whereas USP7 ^C223S^ did not exhibit this effect, confirming that USP7 possesses deubiquitinase activity toward NCOA4 (Fig. 5E). Immunofluorescence microscopy showed colocalization of NCOA4 and USP7, with increased overlap following LPS stimulation (Fig. 5F), consistent with structural docking predictions of a stable USP7–NCOA4 complex (docking score: −270.88; confidence score: 0.9182; Fig. 5G).

*Usp7* silencing in MH-S cells significantly reduced NCOA4 protein levels without altering *Ncoa4* mRNA abundance, confirming post-transcriptional regulation (Fig. 5H–J). Conversely, dose-dependent overexpression of Myc-USP7 gradually increased NCOA4 protein levels without altering *Ncoa4* mRNA levels, further confirming post-translational regulation (Fig. 5K–M). CHX chase experiments revealed that *Usp7* knockdown accelerated NCOA4 protein turnover, while USP7 overexpression extended NCOA4 half-life (Fig. 5N–Q). Importantly, treatment with 3-MA rescued the NCOA4 degradation caused by *Usp7* silencing (Fig. 5R and S), indicating that USP7 stabilizes NCOA4 by inhibiting autophagic degradation. These data confirm that USP7, acting as a deubiquitinase, stabilizes NCOA4 by removing K63-linked ubiquitin chains and suppressing autophagic flux.

### 2.6 S100A9 functions as a key scaffold to facilitate USP7-mediated NCOA4 stabilization

Having established that both S100A9 and USP7 interact with and stabilize NCOA4 by attenuating its K63-linked ubiquitination, we hypothesized that S100A9 acts as a molecular scaffold bridging USP7 and NCOA4.

**Fig. 6:**
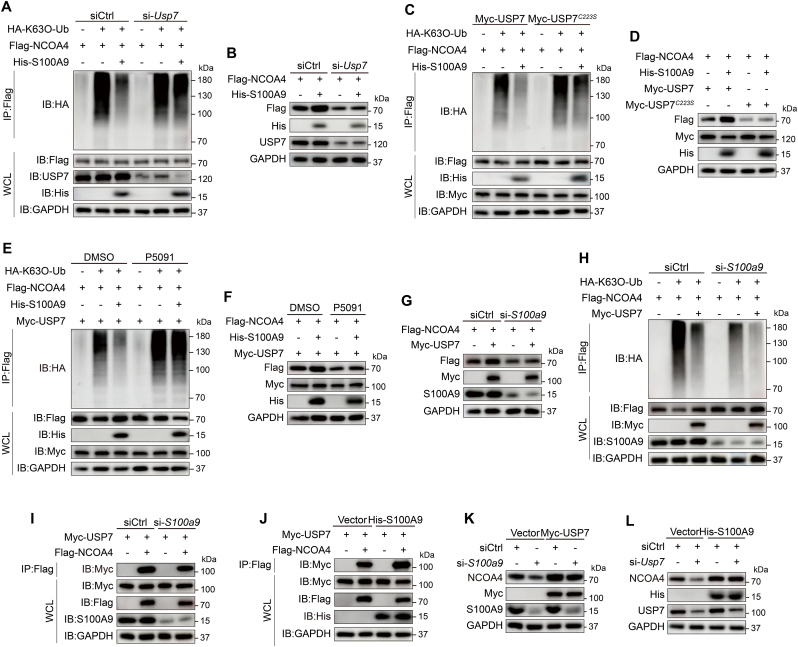
**S100A9 functions as a key scaffold to facilitate USP7-mediated NCOA4 deubiquitination and stabilization. (A)** HA-K63O-Ub, Flag-NCOA4, and His-S100A9 were co-transfected into control or *Usp7*-knockdown HEK-293T cells, lysates were immunoblotted and immunoprecipitated with anti-Flag. **(B)** HEK-293T cells were transfected with HA-K63O-Ub, Flag-NCOA4, and His-S100A9 plasmids, together with either si-Ctrl or si-*Usp7*. Whole-cell lysates were then analyzed by immunoblotting. **(C)** HA-K63O-Ub, Flag-NCOA4, His-S100A9, and either Myc-USP7 or Myc-USP7^C223S^ were co-transfected into HEK-293T cells, lysates were immunoblotted and immunoprecipitated with anti-Flag. **(D)** HEK-293T cells were transfected with Flag-NCOA4 and His-S100A9 plasmids in the presence of either Myc-USP7 or Myc-USP7^C223S^. Whole-cell lysates were then analyzed by immunoblotting. **(E)** Ubiquitination assay with HA-K63O-Ub, Flag-NCOA4, His-S100A9, and Myc-USP7, with cells treated with DMSO or the USP7 inhibitor P5091 (10 μM). **(F)** Immunoblot of Flag-NCOA4, His-S100A9, and Myc-USP7 in WCL of cells treated with DMSO or P5091. **(G)** Immunoblot showing the effect of siCtrl or si-*S100a9* on Flag-NCOA4 and Myc-USP7 protein levels in HEK-293T cells. **(H)** Ubiquitination assay with HA-K63O-Ub, Flag-NCOA4, and Myc-USP7 in siCtrl or si-*S100a9* cells. **(I** and **J)** Co-IP analysis of the interaction between Myc-USP7 and Flag-NCOA4 in HEK-293T cells following si-*S100a9* (I) or His-S100A9 (J). Lysates were immunoprecipitated with an anti-Flag antibody. **(K** and **L)** Immunoblot analysis of NCOA4 in MH-S cells transfected with si-*S100a9* and/or Myc-USP7 (K), and in cells transfected with si-*Usp7* and/or His-S100A9 (L).

To test this hypothesis, we first examined whether S100A9 relies on the deubiquitinase activity of USP7 to exert its stabilizing effect. *In vitro* ubiquitination and Western blot assays revealed that while S100A9 overexpression robustly reduced the K63-linked ubiquitination of NCOA4 and increased its protein abundance, these effects were completely abolished by *Usp7* knockdown (Fig. 6A and B). Similarly, co-transfection with the catalytically inactive USP7^C223S^ mutant failed to support S100A9-mediated NCOA4 stabilization, confirming the strict requirement for USP7 enzymatic activity (Fig. 6C and D). This dependency was further validated using the specific USP7 inhibitor P5091, which effectively neutralized the S100A9-induced reduction in K63-ubiquitination and the subsequent NCOA4 accumulation (Fig. 6E and F).

Next, we investigated whether USP7 requires S100A9 to target NCOA4. Strikingly, while USP7 overexpression normally decreases K63-linked polyubiquitination of NCOA4 and increases its protein levels, *S100a9* knockdown largely neutralized these USP7-mediated effects (Fig. 6G and H). To directly assess the scaffolding role of S100A9, we performed Co-IP assays to evaluate the binding affinity between USP7 and NCOA4. Indeed, *S100a9* knockdown significantly weakened the physical interaction between Myc-USP7 and Flag-NCOA4 (Fig. 6I), whereas His-S100A9 overexpression markedly enhanced their association (Fig. 6J).

Finally, rescue experiments confirmed the epistatic relationship within this axis. USP7 overexpression successfully rescued the loss of NCOA4 induced by *S100a9* knockdown (Fig. 6K). Conversely, S100A9 overexpression could not restore NCOA4 protein levels when *Usp7* was silenced (Fig. 6L). Collectively, these findings demonstrate that S100A9 functions as an essential molecular scaffold that recruits USP7 to NCOA4, thereby facilitating USP7-mediated deubiquitination and subsequent NCOA4 stabilization.

### 2.7 USP7-mediated deubiquitination of NCOA4 at K42 and K181 residues

**Fig. 7:**
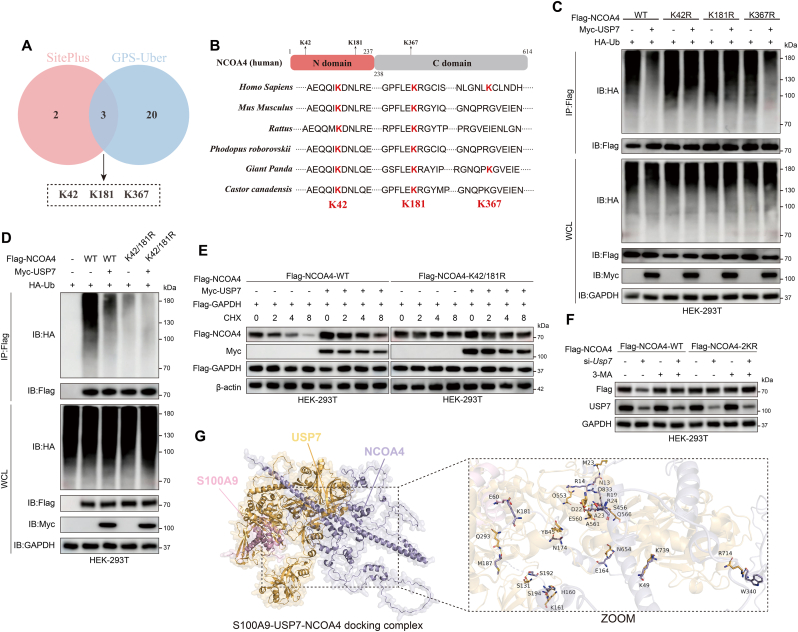
**USP7 mediates deubiquitination of NCOA4 at K42 and K181 residues. (A)** Venn diagram showing the intersection of ubiquitination site predictions from PhosphoSitePlus (pink) and GPS-Uber (blue) tools for NCOA4. Three overlapping candidate sites (K42, K181, K367) were identified. **(B)** Sequence alignment demonstrating the evolutionary conservation of the K42, K181, and K367 residues in NCOA4 across various mammalian species. **(C)** Ubiquitination assay in HEK-293T cells co-expressing HA-Ub, Myc-USP7, and Flag-NCOA4 (WT, K42R, K181R, or K367R single mutants). Immunoprecipitation with anti-Flag. **(D)** Ubiquitination assay comparing Flag-NCOA4 WT versus K42/181R double mutant with Myc-USP7 and HA-Ub. **(E)** CHX chase assay in HEK-293T cells expressing Flag-NCOA4-WT or Flag-NCOA4-K42/181R, with or without Myc-USP7. Flag-GAPDH served as an additional normalization control. Cells were harvested at the indicated time points (0, 2, 4, 8 h) after CHX (100 μg/mL) addition. **(F)** Immunoblot of Flag-NCOA4-WT or Flag-NCOA4-2 KR (K42/181R) in HEK-293T cells transfected with si-*Usp7* and/or treated with 3-MA. USP7 and GAPDH are shown. **(G)** Molecular docking model of the ternary S100A9 (pink)–USP7 (gold)–NCOA4 (purple) complex. The dashed box indicates the interaction interface; the zoomed panel highlights key contact residues including K181 and surrounding amino acids from each protein.

To determine the precise molecular target of USP7 on NCOA4, we sought to identify the specific lysine (K) residues subject to deubiquitination. By cross-referencing data from the PhosphoSitePlus database with predictions from the GPS-Uber algorithm, we identified three highly probable candidate residues: K42, K181, and K367 (Fig. 7A). Subsequent evolutionary conservation analysis revealed that while K42 and K181 are strictly conserved across all examined mammalian species, the conservation of K367 is significantly lower. Although K367 remains highly conserved in primates and certain higher mammals, it exhibits a degree of evolutionary divergence in rodents (Fig. 7B). To determine which of these sites are strictly required for USP7-mediated deubiquitination, we generated Flag-tagged NCOA4 single-point mutants by replacing each lysine with arginine (K42R, K181R, and K367R). Cell-based ubiquitination assays demonstrated that mutating either K42 or K181 partially diminished the basal polyubiquitination of NCOA4 and markedly blunted the deubiquitinating effect of Myc-USP7, whereas the K367R mutation responded similarly to WT NCOA4 (Fig. 7C).

Given that K42 and K181 both contributed to the ubiquitination status, we generated a double mutant (K42/181R, or 2 KR). Strikingly, the NCOA4-2 KR double mutant exhibited a dramatic reduction in baseline K63-linked polyubiquitination and rendered the protein almost completely insensitive to USP7-mediated deubiquitinating activity (Fig. 7D). Consistent with the loss of these ubiquitination sites, CHX chase assays showed that the NCOA4-2 KR mutant had a significantly prolonged half-life compared with NCOA4-WT, and its stability could not be further enhanced by USP7 overexpression (Fig. 7E). Moreover, the NCOA4-2 KR mutant was largely resistant to Usp7 knockdown-induced degradation, maintaining stable protein levels even without USP7-mediated protection (Fig. 7F). Finally, molecular docking of the ternary S100A9–USP7–NCOA4 complex revealed the structural basis by which S100A9 scaffolding positions USP7 in optimal proximity to the K181 site interface on NCOA4, with an extensive network of contact residues spanning all three proteins (Fig. 7G). This structural model is consistent with a mechanism in which S100A9 scaffolding is required to orient USP7 catalytic activity toward K42 and K181, enabling efficient deubiquitination and stabilization of NCOA4. Taken together, these data identify K42 and K181 as the principal K63-ubiquitination sites on NCOA4 that are governed by the S100A9–USP7 scaffolding axis, and whose deubiquitination is essential for NCOA4 stabilization, ferritinophagy receptor function, and downstream ferroptotic signaling.

### 2.8 Pharmacological inhibition of USP7 attenuates sepsis-associated acute lung injury by disrupting the S100A9-NCOA4 axis

**Fig. 8:**
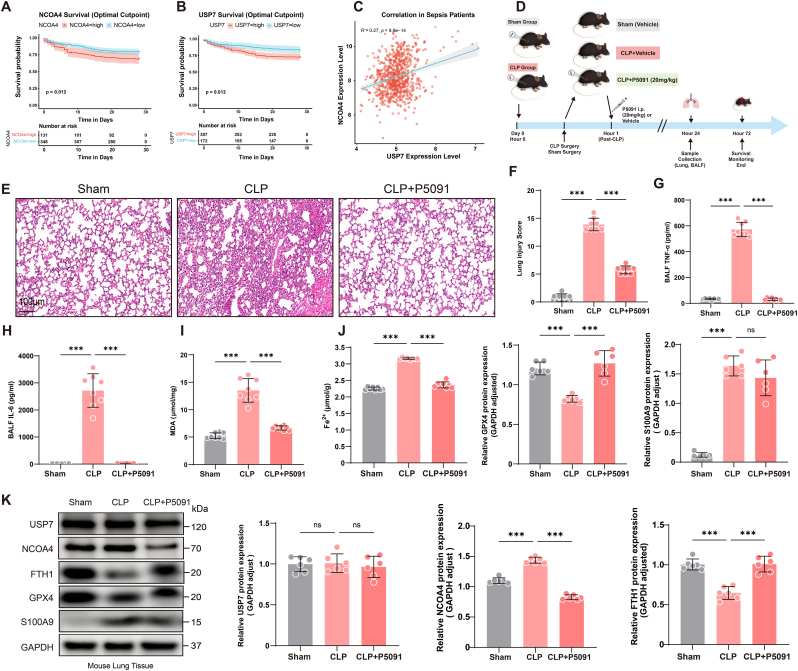
**Pharmacological inhibition of USP7 attenuates sepsis-associated acute lung injury and ferroptosis. (A** and **B)** Kaplan-Meier survival curves depicting the survival probability of sepsis patients stratified by high versus low expression of NCOA4 (A) and USP7 (B) based on optimal cutpoints. **(C)** Correlation analysis between USP7 and NCOA4 expression levels in sepsis patients. **(D)** Schematic representation of the in vivo experimental design, including CLP surgery and P5091 (20 mg/kg) administration. **(E)** Representative H&E-stained lung sections from Sham, CLP + Vehicle, and CLP + P5091 mice. Scale bar, 100 μm. **(F)** Lung injury scores from groups in (E) (n = 8). **(G** and **H)** BALF concentrations of TNF-α (G) and IL-6 (H) measured by ELISA (n = 6). **(I** and **J)** Lung tissue MDA content (I) and labile Fe^2+^ levels (J) in the indicated groups (n = 8). **(K)** Representative immunoblots of USP7, NCOA4, FTH1, GPX4, and S100A9 in lung tissue lysates from Sham, CLP + Vehicle, and CLP + P5091 mice (n = 6). Data are presented as mean ± SD. Statistical analysis by one-way ANOVA with Tukey's post hoc test. ∗∗∗*P* < 0.001; ns, not significant.

To establish the clinical translational relevance of the S100A9-USP7-NCOA4 axis, we first evaluated the expression profiles of these genes in sepsis patients using public clinical datasets (GSE65682). Kaplan-Meier survival analyses demonstrated that elevated expression levels of both NCOA4 and USP7 were significantly associated with poorer survival probabilities in sepsis patients (Fig. 8A and B). Furthermore, we identified a significant positive correlation between the expression levels of USP7 and NCOA4 in this clinical cohort (R = 0.27, p = 9.8e-14), corroborating our in vitro mechanistic findings regarding their tight regulatory relationship (Fig. 8C).

**Fig. 9:**
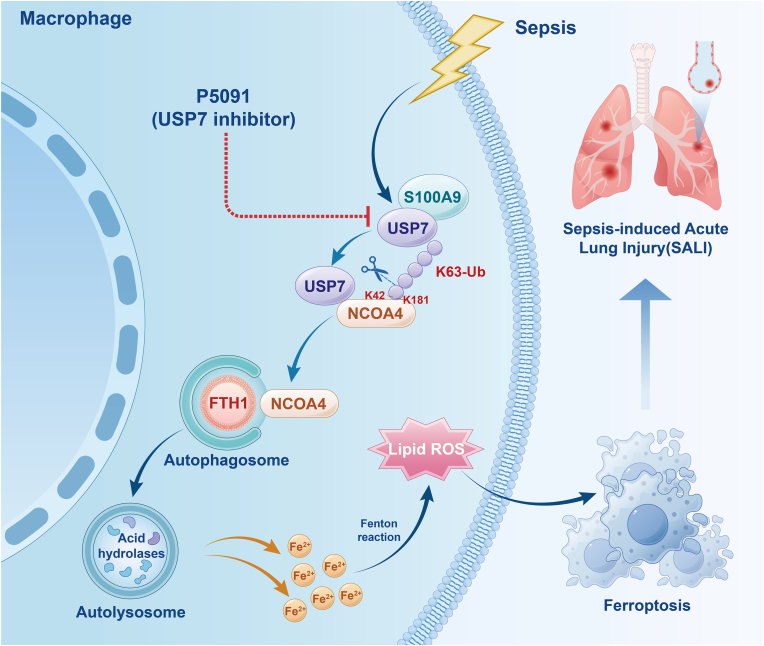
**Schematic model of** S100A9 **scaffolding USP7 and NCOA4 to drive ferritinophagy-mediated ferroptosis in sepsis-induced acute lung injury.** During sepsis, S100A9 expression is upregulated in alveolar macrophages, leading to the recruitment of the deubiquitinase USP7. This S100A9-USP7 complex targets the selective autophagy receptor NCOA4, removing its K63-linked polyubiquitin (K63-Ub) chains and thereby preventing its degradation. The stabilized NCOA4 subsequently binds to FTH1, directing it into autophagosomes. Upon autolysosome formation, FTH1 is degraded by acid hydrolases under low pH conditions, releasing large amounts of Fe^2+^. Intracellular iron overload drives the accumulation of lipid ROS, ultimately leading to ferroptosis in macrophages and exacerbating SALI. Pharmacological inhibition of USP7 using P5091 effectively blocks this pathological cascade, suggesting a potential therapeutic strategy for alleviating SALI.

Encouraged by these clinical correlations, we investigated the therapeutic potential of targeting USP7 in vivo. We administered P5091, a specific pharmacological inhibitor of USP7, to mice subjected to CLP-induced sepsis (Fig. 8D). Histological evaluation revealed that while CLP triggered severe pulmonary structural damage, P5091 treatment effectively preserved lung architecture and significantly reduced lung injury scores (Fig. 8E and F). Consistent with the histological improvement, pharmacological inhibition of USP7 markedly suppressed the excessive release of pro-inflammatory cytokines, including TNF-α and IL-6, in the BALF (Fig. 8G and H).

Importantly, P5091 treatment successfully mitigated CLP-induced pulmonary ferroptosis. The inhibitor significantly diminished the accumulation of MDA and intracellular Fe^2+^ in lung tissues (Fig. 8I and J). Molecularly, in vivo Western blot analysis confirmed that P5091 administration dramatically reduced NCOA4 protein abundance in the lungs of CLP mice without altering the upstream expression of S100A9 or USP7 (Fig. 8K). Consequently, the accelerated degradation of NCOA4 by P5091 effectively prevented ferritinophagy, thereby restoring the critical anti-ferroptotic protein levels of FTH1 and GPX4 (Fig. 8K). Collectively, these findings provide compelling in vivo evidence that pharmacological blockade of USP7 effectively alleviates sepsis-associated acute lung injury by dismantling the S100A9-NCOA4 ferritinophagy axis.

To verify whether USP7 inhibition also disrupts this axis in primary macrophages, we treated primary BMDMs with LPS in the presence or absence of P5091. P5091 did not significantly alter total USP7 protein abundance or S100A9 expression, but markedly reduced LPS-induced NCOA4 accumulation. In parallel, P5091 restored FTH1 and GPX4 expression and attenuated LPS-induced Fe^2+^ accumulation, MDA production, and T-GSH depletion (Fig. S5A–D). These results are consistent with the in vivo findings and support that USP7 inhibition suppresses NCOA4-dependent ferroptosis-associated alterations in primary macrophages. A proposed working model is shown in Fig. 9.

## 3 Discussion

Sepsis-associated acute lung injury is characterized by uncontrolled macrophage activation; however, the mechanisms linking innate immune signaling to regulatory cell death pathways in this context remain poorly understood. In this study, we elucidated a molecular circuit in which S100A9 acts as a protein scaffold to recruit the deubiquitinating enzyme USP7 and direct its catalytic activity toward the ferritinophagy receptor NCOA4. By inhibiting K63-linked polyubiquitination and autophagic degradation of NCOA4, this axis stabilizes NCOA4, expands the labile iron pool, and promotes macrophage ferroptosis, thereby exacerbating SALI. In clinically relevant CLP models, S100A9 knockout or pharmacological inhibition of USP7 disrupts this axis, suppresses ferroptosis signaling, and significantly alleviates lung injury. These findings establish the S100A9–USP7–NCOA4 axis as central to the SALI mechanism and identify USP7 as a viable therapeutic target.

The biological function of S100A9 has been classically attributed to its extracellular role as a DAMP and ligand for TLR4/RAGE. Our findings expand this paradigm by revealing that S100A9 also functions as an intracellular scaffold that directly regulates the post-translational stability of the ferritinophagy receptor NCOA4. This intracellular mechanism is biologically plausible because S100A9 is predominantly enriched in myeloid-lineage cells, particularly neutrophils and monocytes/macrophages, and its expression can be markedly induced during septic inflammation [20]. Multiple mechanisms may contribute to sepsis-induced S100A9 upregulation, including TLR4/RAGE-driven NF-κB/MAPK activation [32], C/EBP-dependent transcriptional regulation [33], hypoxia- or stress-responsive HIF-1α signaling [22], epigenetic remodeling of S100a8/a9-associated regulatory regions, and the recruitment of S100A9-rich inflammatory myeloid populations [34]. These processes may collectively promote de novo intracellular S100A9 accumulation in macrophages during SALI, thereby enabling S100A9 to act beyond its secreted alarmin function.

Multiple lines of evidence support this intracellular scaffolding model. First, endogenous and exogenous co-immunoprecipitation assays, together with molecular docking analyses, confirmed physical interactions between S100A9 and both USP7 and NCOA4. Second, S100A9 deficiency attenuated the interaction between USP7 and NCOA4, whereas S100A9 overexpression enhanced this interaction. Third, the stabilizing effect of S100A9 on NCOA4 depended on the catalytic activity of USP7, as both the catalytically inactive USP7C223S mutant and pharmacological inhibition of USP7 abolished S100A9-mediated NCOA4 accumulation. Fourth, epistasis experiments showed that USP7 overexpression rescued the loss of NCOA4 caused by S100A9 deficiency, whereas S100A9 overexpression could not compensate for USP7 silencing, placing S100A9 upstream of USP7 in this regulatory cascade. Collectively, these findings support a model in which S100A9 functions as a molecular scaffold that facilitates USP7-mediated deubiquitination and stabilization of NCOA4, thereby promoting ferritinophagy and ferroptotic signaling during SALI.

This macrophage-centered model does not exclude potential contributions from other SALI-relevant cell types. Given the abundant expression of S100A9 in neutrophils, it remains possible that related S100A9-dependent programs may operate in neutrophils, although whether they assemble the same S100A9–USP7–NCOA4 complex requires further investigation. In contrast, airway epithelial cells may participate in the broader S100A9-associated inflammatory microenvironment, particularly through responses to extracellular S100A9 or through alternative ferroptosis-regulatory pathways, but the intracellular S100A9–USP7–NCOA4 scaffold axis defined here is primarily supported in macrophages. Notably, a parallel study in hepatocellular carcinoma cells by Zhong et al. [22] demonstrated that HIF-1α-induced intracellular S100A9 can recruit the deubiquitinase USP10 to stabilize PGAM5 and regulate mitochondrial dysfunction, supporting the broader concept that stress-induced intracellular S100A9 may function as a scaffold that spatially organizes deubiquitinase–substrate interactions. Together, these findings suggest that under severe inflammatory stress, S100A9 can transcend its classical extracellular DAMP function and act as an intracellular signaling hub linking innate immune activation to oxidative damage and regulated cell death.

K63-linked ubiquitination acts as a regulatory switch for the autophagy targeting of NCOA4. Our ubiquitination profiling studies revealed that K42 and K181 are the primary K63-linked ubiquitination sites on NCOA4 that mediate its autophagic degradation. This is consistent with the known role of K63-linked polyubiquitin chains as an autophagy-targeting signal, which can be recognized by autophagy receptors such as p62/SQSTM1 and NBR1 [[35], [36], [37]]. The NCOA4-2 KR (K42/181R) double mutant exhibits dramatically reduced baseline ubiquitination, prolonged protein half-life, and near-complete insensitivity to USP7-mediated regulation, confirming that these two sites function cooperatively as the primary regulatory nodes. The evolutionary conservation of K42 and K181 across mammalian species further argues for their functional importance beyond the murine experimental system. Notably, the complexity of NCOA4 stability is further highlighted by a recent study in Cell [38], which demonstrated that the E3 ligase MARCH7 suppresses ferroptosis by targeting NCOA4 for K48-linked ubiquitin-dependent degradation. In contrast, our study identifies a distinct and opposing regulatory mechanism in the highly lethal context of SALI. Specifically, we show that the S100A9–USP7 cascade removes K63-linked polyubiquitin chains from NCOA4, thereby preventing its autophagic degradation. Together, these findings reveal a complex, linkage-specific ubiquitin code governing NCOA4 stability and suggest that targeted deubiquitination of this ferritinophagy receptor determines macrophage fate and exacerbates acute tissue injury. Structurally, our molecular docking model of the ternary S100A9–USP7–NCOA4 complex suggests that S100A9 scaffolding positions the USP7 catalytic domain in optimal proximity to the K181 interface on NCOA4. This structural hypothesis is consistent with the known requirement for DUB positioning by adaptor proteins to achieve efficient and site-specific deubiquitination [39] and identifies K181—and by inference its spatial neighbor K42—as the primary catalytic target within this ternary complex. While co-crystallography or cryo-EM structural determination will ultimately be required to validate the precise interface geometry, the convergent biochemical and in silico data presented here provide a coherent mechanistic framework.

Traditionally, USP7 has been extensively characterized as a high-profile therapeutic target in oncology, where it counteracts ubiquitin-dependent degradation to stabilize diverse oncogenic drivers and immunomodulators [[40], [41], [42], [43], [44]]. While recent studies have expanded its physiological repertoire to include the modulation of cellular oxidative stress [45], the capacity of USP7 to act as a central hub integrating DAMP signaling with the execution of ferritinophagy had not been previously elucidated. Our study now positions USP7 at the intersection of innate immunity and ferroptosis in the context of organ injury. Consistent with prior reports implicating USP7 in sepsis and ferroptosis-related pathways [[24], [25], [26], [27]], we find that genetic or pharmacological USP7 inhibition attenuates SALI. However, we advance this understanding by identifying NCOA4 as a direct deubiquitination substrate of USP7 and by demonstrating that the USP7–NCOA4 regulatory relationship requires S100A9-mediated scaffold assembly. The observation that USP7^C223S^—an active site mutant that retains substrate binding but lacks catalytic activity—fails to stabilize NCOA4 or interact productively with NCOA4 underscores that deubiquitinase activity is indispensable. Importantly, P5091, a small molecule that targets the catalytic domain of USP7 [46], phenocopies genetic *Usp7* knockdown with respect to NCOA4 destabilization, ferroptosis attenuation, and lung protection, suggesting that existing pharmacological tools can be repurposed for SALI therapy. This is further supported by our analysis of the GSE65682 clinical cohort, in which elevated USP7 and NCOA4 expression individually correlated with worse 28-day survival in sepsis patients, and their expression levels were positively correlated, lending human translational support to our mechanistic model.

Ferritinophagy as a nodal mechanism integrating iron, autophagy, and ferroptosis in macrophages. Our data demonstrate that the S100A9–USP7–NCOA4 axis operates through ferritinophagy to amplify labile iron pools and drive lipid peroxidation. This conclusion is supported by multiple lines of evidence: immunofluorescence showing S100A9-dependent enhancement of FTH1–LAMP1 co-localization and lysosomal iron accumulation; NCOA4 rescue experiments demonstrating that *Ncoa4* knockdown abrogates S100A9-driven ferroptosis; and the reversal of these phenotypes by autophagy inhibition with 3-MA or CQ. These findings align with a growing body of literature establishing ferritinophagy as a critical amplifier of ferroptotic cell death in pathological contexts including ischemia-reperfusion injury and cancer [11,47,48]. In the context of SALI, our data suggest that macrophage ferroptosis triggered via the S100A9–NCOA4 axis may act as a feed-forward amplifier of lung injury. This model is consistent with the marked reductions in BALF pro-inflammatory cytokines observed in S100A9-deficient or P5091-treated mice, which likely reflect both the direct anti-inflammatory effects of reduced macrophage ferroptosis and secondary dampening of the inflammatory cascade.

The identification of P5091 as an effective countermeasure against SALI in the CLP model carries immediate translational significance. USP7 inhibitors have been extensively optimized for oncological applications [24], and several compounds have demonstrated acceptable safety profiles in preclinical models, providing a foundation for repurposing in sepsis. Importantly, P5091 did not alter S100A9 or USP7 protein levels in vivo, confirming that its protective effects are mediated through NCOA4 destabilization and subsequent ferritinophagy blockade rather than through broad suppression of upstream inflammatory signals. This mechanistic specificity may limit off-target toxicity compared with broad immunosuppressive strategies, which have historically failed in sepsis clinical trials [49]. Future studies should evaluate the therapeutic window of P5091 in models with defined infection parameters, assess potential interactions with antibiotic regimens, and explore whether combining USP7 inhibition with GPX4 activators or iron chelation offers additive benefit.

Several limitations warrant acknowledgment. First, although our revised study now includes validation in primary BMDMs, direct confirmation in patient-derived alveolar macrophages remains an important future direction. Such studies will require standardized BALF collection from septic patients, sufficient viable macrophage recovery, and careful management of clinical heterogeneity. Second, while our data support a macrophage-centered mechanism, whether neutrophils or airway epithelial cells engage related S100A9-dependent programs during SALI remains unresolved. Future studies using cell-type-specific genetic models, purified neutrophils, airway epithelial cells, and human BALF-derived macrophages will be needed to define the cellular distribution and functional contribution of this pathway in greater detail. Third, although molecular docking provides a structural hypothesis for the ternary S100A9–USP7–NCOA4 complex, experimental structural determination by cryo-EM or X-ray crystallography will ultimately be required to validate the precise interface geometry. Fourth, the GSE65682 dataset is cross-sectional, and the correlative survival analyses, while hypothesis-generating, cannot establish causality. Prospective validation in larger, ethnically diverse sepsis cohorts with standardized sample collection would be valuable. Finally, although our study clearly demonstrated that FTH1 is degraded via an NCOA4-mediated autophagy pathway and promotes the release of free iron, the focus was primarily on the upstream regulators of NCOA4 stability (S100A9 and USP7). Our study did not explore in depth the potential compensatory regulatory mechanisms governing FTH1 transcription or translation in response to acute fluctuations in iron levels during sepsis. Future studies should integrate FTH1 and a broader range of systemic iron metabolism biomarkers to construct a more comprehensive clinical picture of iron metabolism dysregulation in the context of human sepsis.

In summary, we have identified a previously unrecognized molecular axis in which the DAMP protein S100A9 functions as an intracellular scaffold to recruit USP7 to NCOA4, preventing K63-linked autophagic targeting of the ferritinophagy receptor and thereby sustaining a ferritin-degradation–iron liberation–lipid peroxidation cascade that drives macrophage ferroptosis and exacerbates SALI. Pharmacological targeting of USP7 with P5091 effectively dismantles this axis, offering a mechanistically precise and pharmacologically tractable strategy for the treatment of sepsis-associated lung injury.

## 4 Materials and methods

### 4.1 Bioinformatics analysis

Clinical data and pre-processed microarray datasets of sepsis patients (GSE65682) were retrieved from the Gene Expression Omnibus (GEO) database. Probe IDs were mapped to gene symbols, and mean values were calculated for multiple probes targeting the same gene. To investigate co-expression, USP7 and NCOA4 expression profiles were extracted for Pearson correlation analysis. To assess clinical prognosis, optimal cut-off values for gene expression were determined using the surv_cutpoint function in R. Kaplan-Meier survival curves were subsequently generated to evaluate 28-day mortality, with statistical differences assessed using the log-rank test. All statistical analyses and visualizations were performed using R software (version 4.5.3), and a two-sided P-value <0.05 was considered statistically significant.

### 4.2 Animal models and treatments

The WT C57BL/6J mice were purchased from Vital River Laboratories (Beijing, China), and the S100a9^em1/Cya^ (S100a9-KO) mice (Strain S–KO-16221) with a C57BL/6J background were obtained from Cyagen (Suzhou, China). All animals were housed in SPF-grade animal rooms with a 12-h light/dark cycle, maintained at a temperature of 20-24 °C and a relative humidity of 45%-65%. We established a polymicrobial sepsis model using CLP. Briefly, we anesthetized mice via intraperitoneal sodium pentobarbital injection, ligated the distal cecum, and punctured it twice with a 21-gauge needle to extrude a small amount of feces. Sham mice underwent laparotomy without ligation or puncture. For rescue and intervention experiments, we intraperitoneally injected recombinant His-S100A9 protein (20 μg/mouse; Biosune, Shanghai, China) or the USP7 inhibitor P5091 (20 mg/kg, MCE, Shanghai, China) post-CLP. We euthanized mice at 24 h post-surgery and collected BALF and lung tissues for downstream analysis. Mice were randomly assigned to different groups.

### 4.3 Cell culture and reagents

MH-S (CL-0597) and HEK-293T (CL-0005) cell lines were obtained from Procell (Wuhan, China). Both cell lines were authenticated via Short Tandem Repeat (STR) profiling and confirmed to be free of mycoplasma contamination. MH-S cells were maintained in RPMI-1640 medium, while HEK-293T cells were cultured in high-glucose DMEM. Both media were supplemented with 10% fetal bovine serum (FBS; Lonsera, China) and 1% penicillin-streptomycin (P/S; Cytiva, USA). All cells were incubated at 37 °C in a humidified atmosphere containing 5% CO_2_.

### 4.4 Isolation and culture of bone marrow-derived macrophages

Primary bone marrow-derived macrophages (BMDMs) were isolated from WT and S100a9-deficient mice. Briefly, bone marrow cells were flushed from the femurs and tibias under sterile conditions, passed through a 70-μm cell strainer, and collected by centrifugation. After red blood cell lysis, the cells were cultured in RPMI-1640 medium supplemented with 10% fetal bovine serum, 1% penicillin-streptomycin, and recombinant mouse M-CSF to induce macrophage differentiation. Fresh differentiation medium was added during culture, and mature adherent BMDMs were obtained on day 7. For subsequent experiments, BMDMs were replated and stimulated with LPS in the presence or absence of P5091 as indicated. Cells were then collected for western blotting, biochemical assays, and flow cytometric analysis. For BMDM validation experiments, Fe^2+^, MDA, T-GSH, total ROS, and lipid ROS were measured using the same commercial kits, staining protocols, and analysis procedures as those used for MH-S cells unless otherwise specified.

### 4.5 Iron quantification

Ferrous iron (Fe^2+^) concentrations in MH-S cells, primary BMDMs, and mouse lung tissues were determined using specific fluorometric (Elabscience, E-BC-F101, Wuhan, China) and colorimetric (Solarbio, BC5415, Beijing, China) assay kits, respectively, according to the manufacturers' instructions. Briefly, the samples were processed in designated extraction buffers. Cellular Fe^2+^ levels were subsequently quantified by measuring fluorescence at an excitation wavelength of 542 nm and an emission wavelength of 575 nm, whereas tissue Fe^2+^ levels were quantified by measuring absorbance at 593 nm using a microplate reader. All iron concentrations were normalized to total protein content, which was quantified using a BCA Protein Assay Kit (Beyotime, P0010, Shanghai, China).

### 4.6 Determination of lipid ROS and lipid peroxidation

Total intracellular ROS and lipid peroxidation levels were assessed in MH-S cells and primary BMDMs using DCFH-DA (Beyotime, S0034S) and BODIPY 581/591 C11 (MCE, HY-D1301) fluorescent probes, respectively. Fluorescence images were acquired using an EVOS M7000 imaging system (Thermo Fisher Scientific, USA). BMDMs were processed using the same staining and flow cytometry procedures. For quantitative analysis, the fluorescence intensity of the cells was measured using a CytoFLEX flow cytometer (Beckman Coulter, USA), and the resulting data were analyzed using FlowJo software (Tree Star, USA).

For the biochemical evaluation of cellular redox status and lipid peroxidation, the levels of MDA and T-GSH in MH-S cells, primary BMDMs, and mouse lung tissue homogenates were quantified using specific colorimetric assay kits (Elabscience and Beyotime, China) according to the manufacturers’ instructions. Absorbance was measured using a microplate reader. For cultured cells, including MH-S cells and BMDMs, the same assay procedures were applied, and results were normalized to cell number or total protein concentration as indicated in the corresponding figure legends. For lung tissue samples, values were normalized to total protein concentration determined using a BCA Protein Assay Kit. (Beyotime, P0010).

### 4.7 Reagents and antibodies

LPS (*E-coli*, O55: B5, L2880) was purchased from Sigma-Aldrich (St Louis, USA). MG132 (HY-13259), Cycloheximide (HY-12320), Chloroquine (HY-17589A), 3-Methyladenine (HY-19312), Rapamycin (HY-10219) were purchased from MCE (Shanghai, China). Anti-S100A9 (73425S) was obtained from Cell Signaling Technology (MA, USA). Anti-ACSL4 (22401-1-AP), anti-GPX4 (67763-1-Ig), anti-NCOA4 (10968-1-AP), anti-USP7 (66514-1-Ig), anti-p62/SQSTM1 (18420-1-AP), anti-LC3b (14600-1-AP), anti-GAPDH (60004-1-Ig), anti-HA (51064-2-Ap), anti-His (66005-1-Ig), anti-DDDDK/Flag (66008-3-Ig), anti-Myc (10828-1-AP) were purchased from Proteintech Group (Wuhan, China). Anti-FTH1 (18M69L95) was purchased from Epizyme Biotech (Shanghai, China).

### 4.8 Plasmid construction and transfection

His-S100A9, Myc-USP7, Myc-USP7^*C223S*^ and Flag-GAPDH were purchased from Abiotech (Jinan, China). Plasmids for HA-tagged WT ubiquitin or its mutants (K27, K48, K63, K63R) and Flag-NCOA4, Flag-NCOA4-K42R, Flag-NCOA4-K181R, Flag-NCOA4-K42/181R (2 KR) were purchased from BioSune Biotechnology (Shanghai, China). All plasmid constructs were verified by DNA sequencing. Plasmids were transiently transfected into cells using Lipofectamine 3000 reagent (Invitrogen, USA) according to the manufacturer's instructions.

### 4.9 RNA extraction and quantitative real-time PCR

Total RNA was extracted using the Fastagen RNA Extraction Kit (Shanghai, China). Complementary DNA (cDNA) was synthesized using the HiScript III RT SuperMix for qPCR (+gDNA wiper) (Vazyme, R323, Nanjing, China) according to the manufacturer's instructions. Quantitative real-time PCR (RT-qPCR) was performed on a CFX96 Touch Real-Time PCR Detection System (Bio-Rad, Hercules, CA, USA) using a SYBR Green master mix (Vazyme; Q713). Target gene expression was normalized to Gapdh. All samples were analyzed in triplicate, and relative mRNA levels were calculated using the 2−ΔΔCt method. Primer sequences are provided in Supplementary Table S1.

### 4.10 Western blot analysis

Briefly, proteins were extracted from cells and tissues using RIPA buffer (Epizyme Biotech, PC102) containing protease inhibitors (Servicebio, G2006-250UL), and concentrations were quantified via a BCA assay (Sevenbio, SW101). Equal amounts of protein were separated by SDS-PAGE and transferred to PVDF membranes (Millipore, C3117, USA). After blocking with 5% BSA (Seven Biotech, SO110, Beijing, China) for 1 h at room temperature, the membranes were incubated with specific primary antibodies overnight at 4 °C, followed by incubation with HRP-conjugated secondary antibodies (ZSGB-bio, ZB-2301 and ZB-2305, Beijing, China) for 1 h at room temperature. Chemiluminescence was visualized using Omni-ECL Western Blotting Substrate (Epizyme Biotech, SQ203) on an Amersham ImageQuant 800 system (Cytiva, USA). Densitometric analysis of the blots was performed using ImageJ software (National Institutes of Health, NIH, USA).

### 4.11 Co-immunoprecipitation (Co-IP) assay

As previously described, treated cells were lysed in Pierce lysis buffer (25 mM Tris-HCl, pH 7.4, 150 mM NaCl, 1 mM EDTA, 1% NP-40, and 5% glycerol; Thermo Fisher Scientific, 87788) supplemented with a protease inhibitor cocktail (Servicebio, G2006-250UL). Cell lysates were incubated with the indicated primary antibodies overnight at 4 °C with rotation. Subsequently, Protein A/G magnetic beads, which had been pre-washed with lysis buffer, were added to the mixture and incubated for another 4 h. After washing the beads five times with lysis buffer, the immunoprecipitated proteins were eluted and subjected to Western blot analysis.

### 4.12 RNA interference

MH-S and HEK-293T cells were seeded in 6-well plates and cultured for 12 h. After incubation, the cells were transfected with 100 nM siRNA using Lipo3000 reagent (Invitrogen, USA). The siRNAs were synthesized by BioSune Biotechnology (Shanghai, China), and the sequences of the siRNAs used are provided in Supplementary Table S2.

### 4.13 Immunofluorescence and fluorescence microscopy

For live-cell imaging of lysosomal Fe^2+^, MH-S cells were co-incubated with LysoTracker Green (Invitrogen, A66438) and FerroOrange (CST, 36104) for 30 min at 37 °C, followed by nuclear staining with Hoechst 33342 (Sigma-Aldrich, 94403). For protein colocalization analysis, MH-S cells were fixed, permeabilized, and blocked with 5% bovine serum albumin (BSA). The cells were subsequently incubated overnight at 4 °C with primary antibodies against LAMP1 (1:100; Santa Cruz, sc-20011) and FTH1 (1:100; Epizyme, R013827). After washing, the cells were incubated with fluorophore-conjugated secondary antibodies (Invitrogen, A-11072 and A24920), and the nuclei were counterstained with DAPI (Abcam, ab104139). To resolve host-species cross-reactivity during the co-staining of S100A9 (1:1000; Proteintech, 26992-1-AP) and NCOA4 (1:2000; Proteintech, 10968-1-AP), a multiplex tyramide signal amplification (TSA) strategy was employed using iF488 and iF647 tyramide (Servicebio, G1233). Between sequential TSA cycles, the primary and secondary antibody complexes were stripped using an elution buffer (Servicebio, G1266) to ensure signal specificity. Finally, the subcellular localization of the labile iron pool and specific proteins was visualized and acquired using a Nikon A1R HD25 confocal microscope (Nikon, Japan).

### 4.14 ELISA assay

The concentrations of pro-inflammatory cytokines, including TNF-α, IL-6 (Epizyme, China), and IL-1β (ELK Biotechnology, China), in the BALF supernatants were quantified using specific ELISA kits according to the manufacturers' protocols. Absorbance was measured at 450 nm using a microplate reader, and cytokine concentrations were calculated based on standard curves generated in parallel.

### 4.15 Hematoxylin-eosin (H&E) staining and lung injury scoring

Lung tissues were fixed in 4% paraformaldehyde (Servicebio, G1101) for over 24 h, embedded in paraffin, and sectioned at a thickness of 4 μm. Following deparaffinization in xylene and rehydration through a graded ethanol series, the sections were stained with Harris hematoxylin for 5 min. After differentiation with 1% acid alcohol and bluing with 0.6% ammonia water, the sections were counterstained with eosin for 2 min. Finally, the slides were dehydrated through graded alcohols, cleared in xylene, and mounted with neutral resin. Images were acquired using a Nikon Eclipse Ci upright optical microscope equipped with a Nikon DS-U3 imaging system (Nikon, Japan).

To evaluate the severity of SALI, lung injury scores were assessed in three randomly selected, non-overlapping fields per section using a 20-point scoring system. The system comprised four parameters, each graded on a scale of 0 to 5: (i) inflammatory cell infiltration, (ii) alveolar edema, (iii) alveolar hemorrhage, and (iv) septal hemorrhage and congestion. The total lung injury score was calculated as the sum of these four parameters, yielding a maximum possible score of 20. All histological evaluations were conducted in a blinded manner to ensure objectivity.

### 4.16 Immunohistochemical (IHC) analyses

Lung tissues were fixed in 4% paraformaldehyde (Servicebio, G1101) for over 24 h, embedded in paraffin, and sectioned at a thickness of 5 μm. Following deparaffinization and rehydration, microwave-assisted heat-induced antigen retrieval was performed using EDTA buffer (pH 9.0). The sections were treated with 3% hydrogen peroxide for 25 min to quench endogenous peroxidase activity, blocked with 3% bovine serum albumin (BSA) for 30 min at room temperature, and subsequently incubated overnight at 4 °C with primary antibodies against S100A9 (1:200; Proteintech; 26992-1-AP), NCOA4 (1:400; Proteintech; 10968-1-AP), ACSL4 (1:100; Proteintech; 22401-1-AP), and GPX4 (1:1000; Proteintech; 67763-1-Ig). After washing with PBS, the sections were incubated with HRP-conjugated secondary antibodies for 50 min at room temperature. Immunoreactivity was visualized using a 3,3′-diaminobenzidine (DAB) substrate. Nuclei were counterstained with Harris hematoxylin, and the slides were mounted with neutral resin. Images were acquired using a Nikon Eclipse Ti-SR inverted microscope equipped with a Nikon DS-U3 imaging system (Nikon, Japan).

### 4.17 Bronchoalveolar lavage fluid (BALF) collection and analysis

BALF was collected via tracheotomy and tracheal cannulation. The lungs were lavaged three times with a total of 3 mL of ice-cold sterile PBS. The recovered fluid was centrifuged at 400 × g for 10 min at 4 °C. The resulting supernatant was collected and stored at −80 °C for subsequent biochemical analysis. The total protein concentration in the supernatant was quantified using a BCA Protein Assay Kit (Beyotime, P0010).

### 4.18 Lung wet/dry ratio

The severity of pulmonary edema was assessed by determining the lung wet-to-dry (W/D) weight ratio. The right lung was excised and immediately weighed to obtain the wet weight (W). Subsequently, the tissue was dried in an oven at 80 °C for 48 h until a constant weight was reached, which was recorded as the dry weight (D). The W/D ratio was then calculated to quantify water accumulation in the lung tissue.

### 4.19 Molecular docking

Molecular docking simulations were performed to predict atomic-level protein interaction interfaces. The HDOCK server was used to predict binding modes between S100A9 and NCOA4, and between the USP7 catalytic domain and NCOA4. The docking results were visualized using PyMOL (Schrödinger), hydrogen bonds and contact networks involving the K42 and K181 residues were analyzed, and docking and confidence scores were evaluated.

## 5 Statistics

All statistical analyses were performed using GraphPad Prism 9 (GraphPad Software, USA). Data are expressed as mean ± standard deviation (SD) from at least three independent biological replicates. Comparisons between two groups were assessed using a two-tailed Student's t-test, and multiple-group comparisons were evaluated by one-way or two-way ANOVA with appropriate post hoc tests. A *P* value < 0.05 was considered statistically significant. Significance levels are denoted as follows: ∗*P* < 0.05, ∗∗*P* < 0.01, ∗∗∗*P* < 0.001, ∗∗∗∗*P* < 0.0001; and ns means no significance.

## 6 Study approval

All animal procedures and experimental protocols in this study were reviewed and approved by the Ethics Review Committee of Shandong First Medical University (approval no. W202411190702).

## Funding

10.13039/501100001809National Natural Science Foundation of China grant 82270093 (YLW).

## CRediT authorship contribution statement

**Yi Wei:** Conceptualization, Data curation, Investigation, Methodology, Software, Validation, Visualization, Writing – original draft, Writing – review & editing. **Angran Gu:** Conceptualization, Data curation, Investigation, Methodology, Validation, Writing – review & editing. **Bailun Wang:** Conceptualization, Investigation, Methodology, Validation, Writing – review & editing. **Yizheng Yang:** Investigation, Methodology, Visualization. **Chang Sun:** Investigation, Methodology, Visualization. **Yi Zhang:** Investigation, Methodology. **Runmeng Liu:** Investigation. **Yichen Wang:** Investigation. **Changping Gu:** Investigation. **Yuelan Wang:** Funding acquisition, Investigation, Methodology, Resources, Writing – review & editing.

## Declaration of competing interest

The authors declare that they have no known competing financial interests or personal relationships that could have appeared to influence the work reported in this paper.

## Data Availability

Publicly available data were obtained from the GEO database under accession number GSE65682. Other data are available from the corresponding author upon reasonable request.
