# Supplementary material for: S100A9 modulates USP7-mediated stabilization of NCOA4 to promote ferroptosis in sepsis-associated acute lung injury
Source: Redox Biol. 2026 Jun 21;95:104271. doi: 10.1016/j.redox.2026.104271 (PMC13316292; doi:10.1016/j.redox.2026.104271)
Supplement: Multimedia component 1 [file mmc1.docx]

Supplementary Materials for

**S100A9 Modulates USP7-Mediated Stabilization of NCOA4 to Promote Ferroptosis in Sepsis-Associated Acute Lung Injury**

Yi Wei and Angran Gu *et al.*

^#^Corresponding author: Yuelan Wang Email: [LXQ9066@163.com](mailto:LXQ9066@163.com)

**This PDF file includes:**

Figures. S1 to S5

Tables S1 to S2


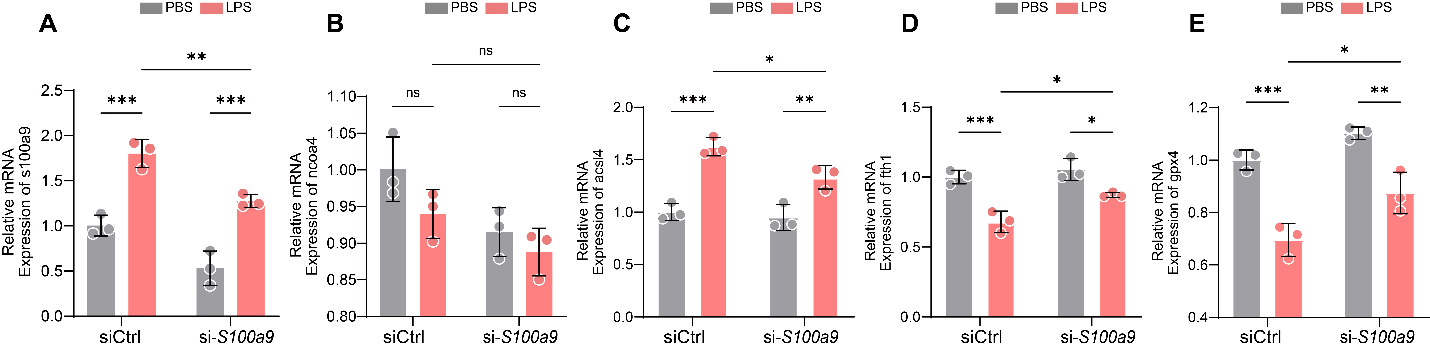


**Fig. S1. S100A9 mediates ferroptosis-related transcriptional shifts without altering *Ncoa4* mRNA expression.** **(A**-**E)** Relative mRNA expression levels of *S100a9* (**A**), *Ncoa4* (**B**), *Acsl4* (**C**), *Fth1* (**D**), and *Gpx4* (**E**) in MH-S macrophages were determined by RT-qPCR. Cells were transfected with either siCtrl or si-*S100a9* prior to stimulation with PBS or LPS. Data are presented as mean ± SD from at least three independent experiments. Statistical significance was assessed by two-way ANOVA with Tukey's post hoc test. **P* < 0.05, ***P* < 0.01, ****P* < 0.001, and ns indicates not significant.


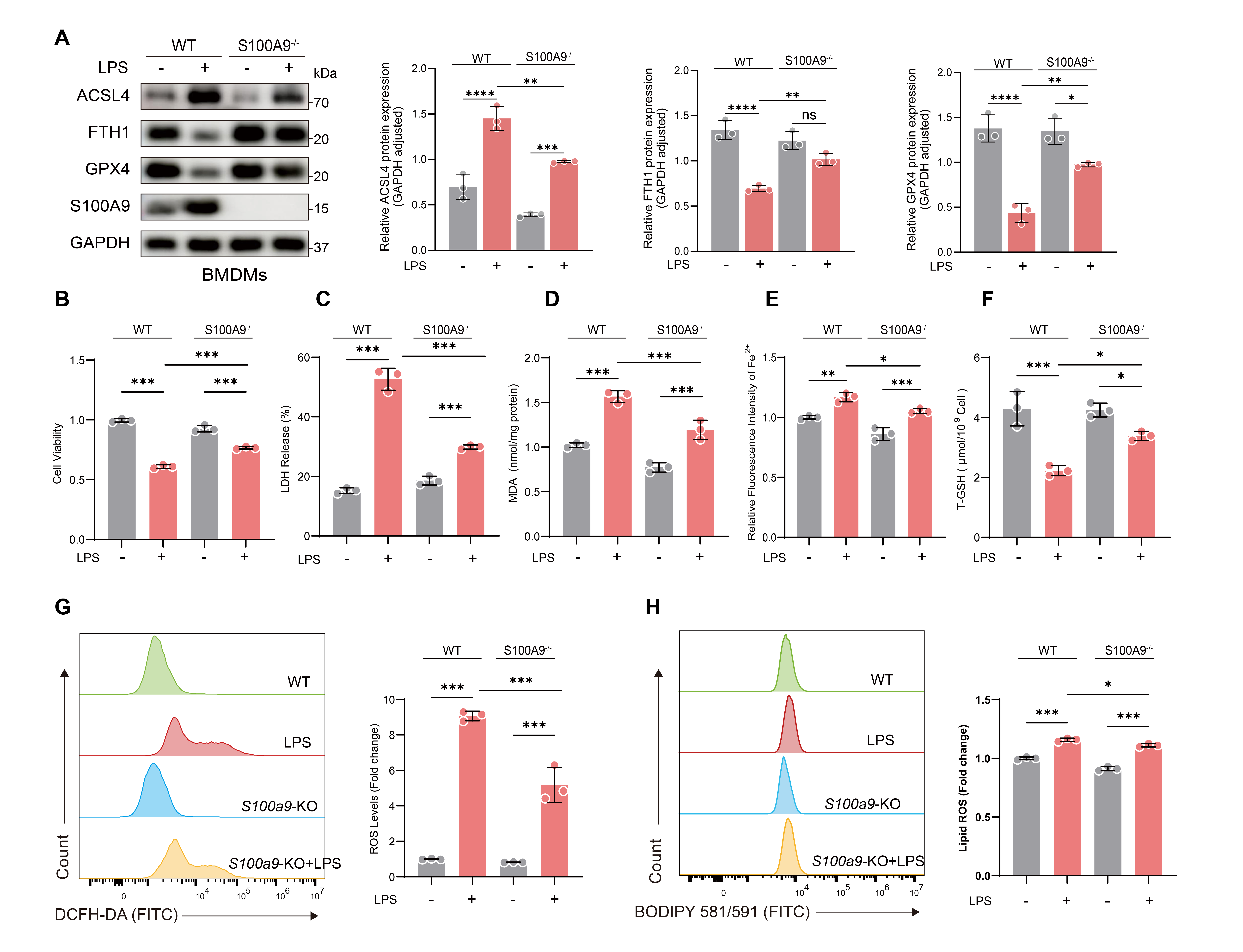


**Fig. S2. S100A9 deficiency attenuates LPS-induced ferroptosis in primary BMDMs.
(A)** Representative immunoblots and quantification of ACSL4, FTH1, GPX4, and S100A9 protein expression in WT and S100a9^-/-^ BMDMs treated with PBS or LPS. GAPDH was used as the loading control. **(B** and **C)** Cell viability (B) and LDH release (C) were measured in WT and S100a9^-/-^ BMDMs across the indicated treatment groups. **(D** to **F)** Quantification of intracellular MDA levels (D), relative fluorescence intensity of ferrous iron (E), and T-GSH levels (F) in BMDMs. **(G)** Representative flow cytometry histograms of DCFH-DA fluorescence and quantification of total ROS levels in BMDMs under the indicated conditions. **(H)** Representative flow cytometry histograms of BODIPY 581/591 staining and quantification of lipid ROS levels. Data are presented as mean ± SD from three independent BMDM preparations. Statistical significance was assessed by ordinary one-way ANOVA with Tukey’s post hoc test. **P* < 0.05, ***P* < 0.01, ****P* < 0.001, and *****P* < 0.0001; ns, not significant.


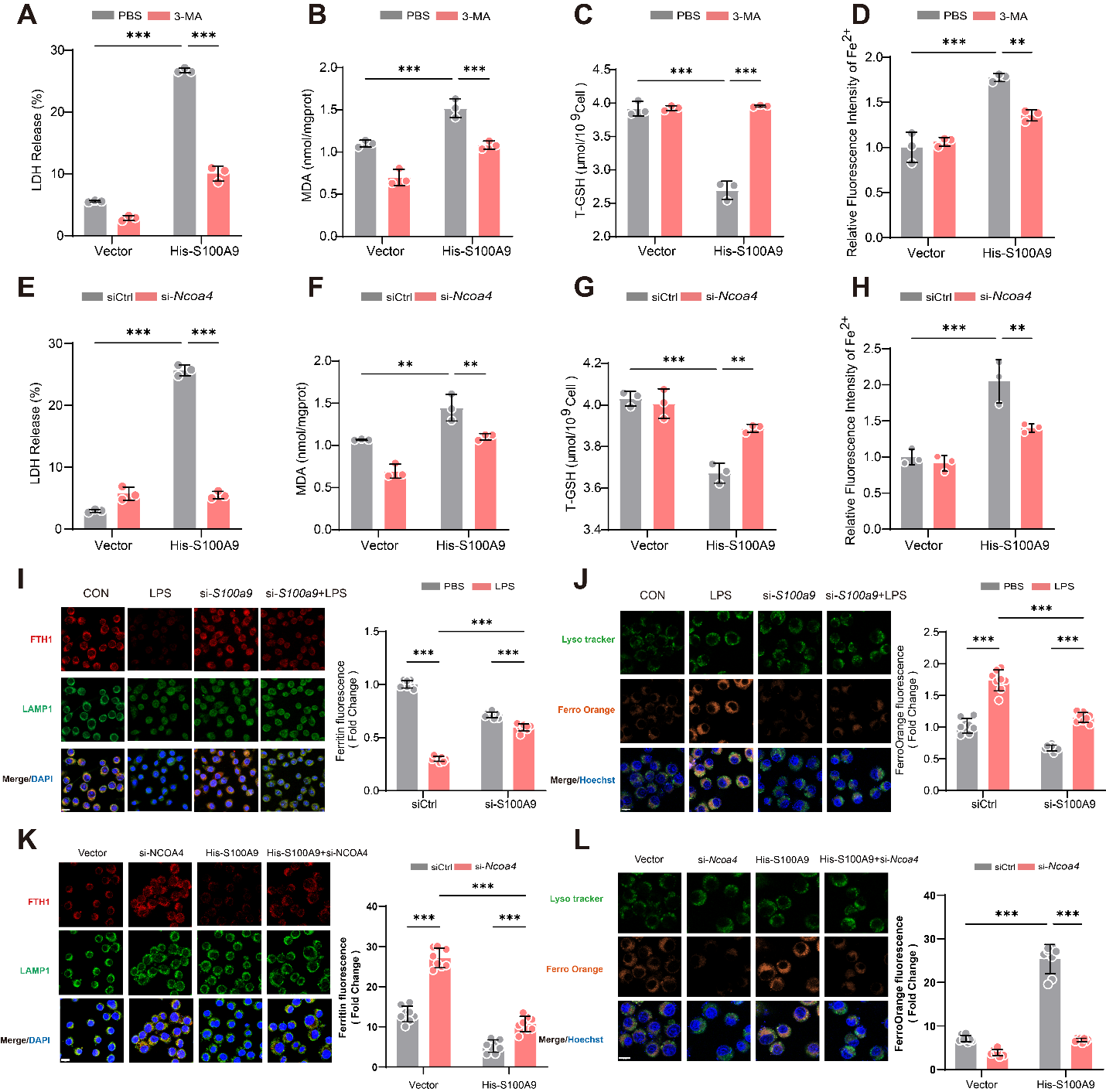
 Fig. S3. S100A9 promotes ferritinophagy and macrophage ferroptosis *in vitro*. (A-D) Quantification of LDH release (A), intracellular MDA (B), T-GSH (C), and relative Fe²⁺ fluorescence intensity (D) in MH-S cells transfected with Vector or His-S100A9 plasmids, with or without 3-MA treatment. (E-H) Measurement of LDH release (E), MDA (F), T-GSH (G), and relative Fe²⁺ fluorescence intensity (H) in MH-S cells cotransfected with Vector or His-S100A9 and siCtrl or si-*Ncoa4*. (I, J) Representative confocal images of FTH1 (red) and LAMP1 (green) colocalization (I) and live-cell imaging of lysosomes (LysoTracker, green) and labile Fe²⁺ (FerroOrange, orange) (J) in MH-S cells treated with PBS or LPS, with or without *S100a9* knockdown. (K, L) Representative confocal images of FTH1/LAMP1 colocalization (K) and live-cell LysoTracker/FerroOrange imaging (L) in MH-S cells with S100A9 overexpression and/or *Ncoa4* knockdown. Scale bars, 10 μm. Data are presented as mean ± SD from at least three independent experiments. Statistical significance was assessed by two-way ANOVA with Tukey's post hoc test. ** *P* < 0.01, *** *P* < 0.001.


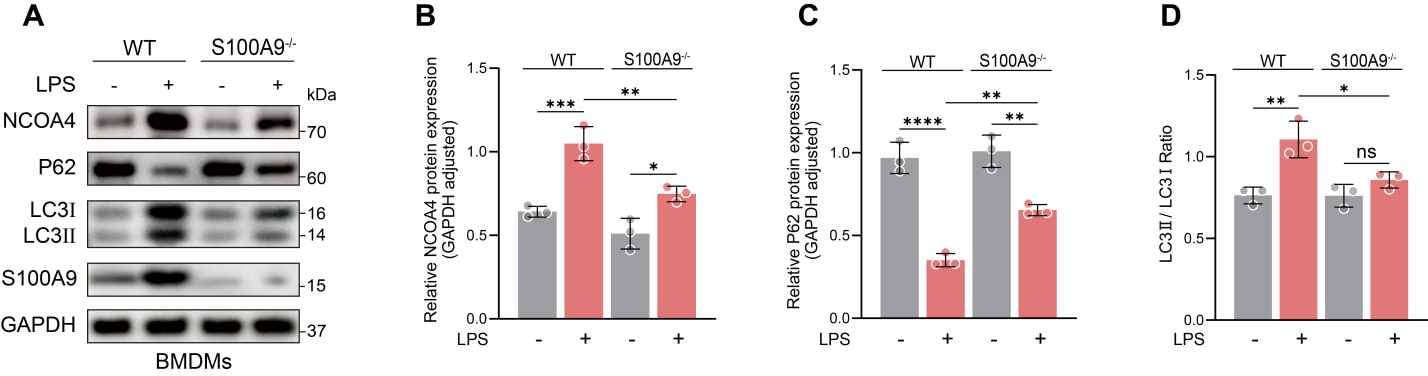


**Fig. S4. S100A9 deficiency suppresses LPS-induced NCOA4 accumulation and ferritinophagy-associated autophagic activation in primary BMDMs.
(A** to **D)** Western blot analysis and corresponding quantification of NCOA4, p62, and LC3 in WT and S100a9^−/−^ BMDMs under the indicated treatment conditions. GAPDH was used as the loading control. Data are presented as mean ± SD from three independent BMDM preparations. Statistical significance was assessed by ordinary one-way ANOVA with Tukey’s post hoc test. **P* < 0.05, ***P* < 0.01, ****P* < 0.001, and *****P* < 0.0001; ns, not significant.


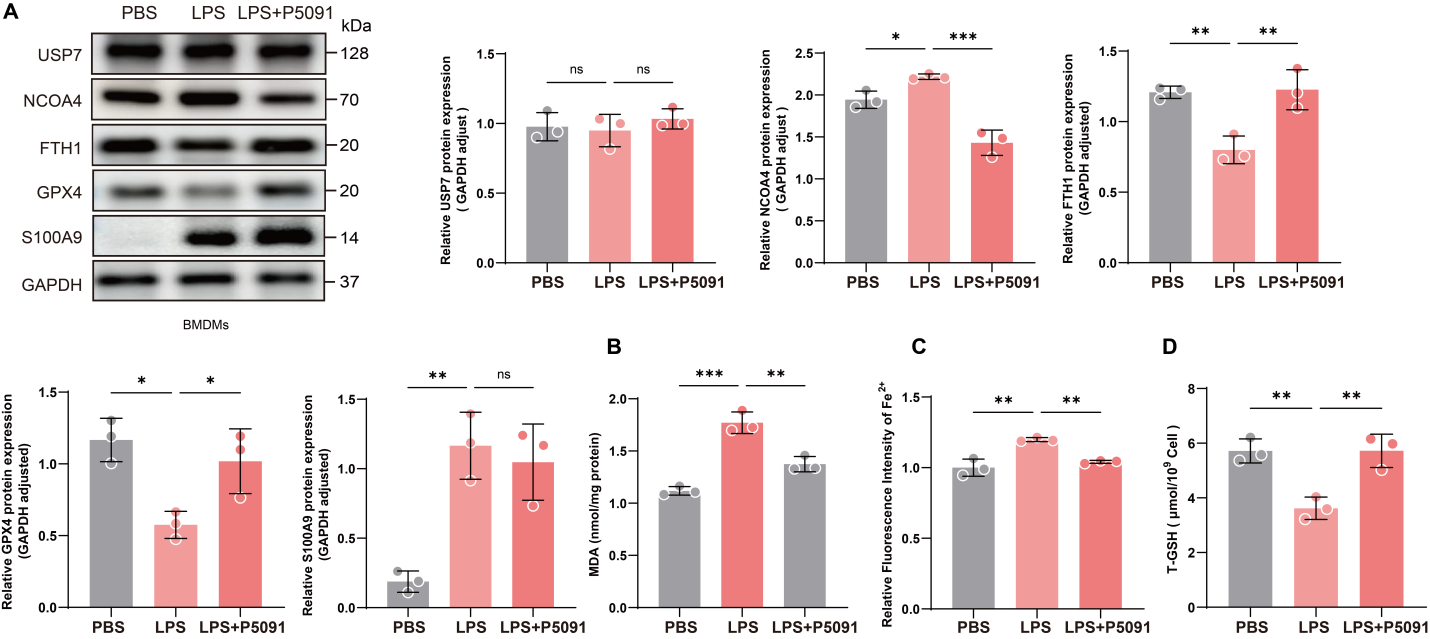


**Fig. S5. USP7 inhibition attenuates NCOA4 accumulation and ferroptosis-associated alterations in LPS-stimulated primary BMDMs.
(A)** Representative immunoblots and corresponding quantification of USP7, NCOA4, FTH1, GPX4, and S100A9 protein expression in primary BMDMs treated with PBS, LPS, or LPS plus P5091. GAPDH was used as the loading control. **(B** to **D)** Quantification of intracellular MDA levels (B), relative fluorescence intensity of ferrous iron (C), and T-GSH levels (D) in primary BMDMs across the indicated treatment groups. Data are presented as mean ± SD from three independent BMDM preparations. Statistical analysis was performed by one-way ANOVA with Tukey’s post hoc test. **P* < 0.05, ***P* < 0.01, ****P* < 0.001; ns, not significant.

Table S1.

**Primer sequences of certain genes used in RT-qPCR**

| **Gene name** | **Sequence (5’-3’)** |
| --- | --- |
| ***mus-S100a9*** |  |
| F | CAGCTGAGCTTTGAGGAGTGT |
| R | TCCCACAGCCTTTGCCATGA |
| ***mus-Gpx4*** |  |
| F | GTGTAAATGGGGACGATGCC |
| R | ACCACGCAGCCGTTCTTATC |
| ***mus-Ncoa4*** |  |
| F | TATCCAGGTGCCAGAGCAGA |
| R | GGATAAGCCAGTCCTGTGGG |
| ***mus-Fth1*** |  |
| F | CAGAACTACCACCAGGACGC |
| R | TCAGAGCCACATCATCTCGG |
| ***mus-Acsl4*** |  |
| F | GCTATGACGCCCCTCTTTGT |
| R | GAATCGGTGTGTCTGAGGGG |
| ***mus-Slc7a11*** |  |
| F | ATACTCCAGAACACGGGCA |
| R | AGGGCTCCAAAAAGTGACAGT |
| ***mus-Usp7*** |  |
| F | GCATACTGGCTATGTCGGCT |
| R | GGGCTTATCGCTGTGCTGTA |
| ***mus-Gapdh*** |  |
| F | TGTCTCCTGCGACTTCAACA |
| R | GGTGGTCCAGGGTTTCTTACT |

Table S2.

**The siRNA sequences targeting indicated genes were listed**

| **Gene name** | **Sequence (5’-3’)** |
| --- | --- |
| **mus-si*S100a9#1*** |  |
| S | GCCCUCAUAAAUGACAUCATT |
| AS | UGAUGUCAUUUAUGAGGGCTT |
| **mus-si*S100a9#2*** |  |
| S | CCUCAUAAAUGACAUCAUGTT |
| AS | CAUGAUGUCAUUUAUGAGGTT |
| **homo-si*S100a9*** |  |
| S | AUGGAGGACCUGGACACAATT |
| AS | UUGUGUCCAGGUCCUCCAUTT |
| **mus-si*Ncoa4*** |  |
| S | GGGCUGAACAGCAAAUUAATT |
| AS | UUAAUUUGCUGUUCAGCCCTT |
| **mus-si*Usp7*** |  |
| S | GCCCAAGUUUGAUAAAGAUTT |
| AS | AUCUUUAUCAAACUUGGGCTT |
| **homo-si*Usp7*** |  |
| S | GCCGACACCAGUACAUAAATT |
| AS | UUUAUGUACUGGUGUCGGCTT |
| **Negative Control** |  |
| S | UUCUCCGAACGUGUCACGUTT |
| AS | ACGUGACACGUUCGGAGAATT |
