# Supplementary material for: S100A9 modulates USP7-mediated stabilization of NCOA4 to promote ferroptosis in sepsis-associated acute lung injury
Source: Redox Biol. 2026 Jun 21;95:104271. doi: 10.1016/j.redox.2026.104271 (PMC13316292; doi:10.1016/j.redox.2026.104271)

Fig.1A

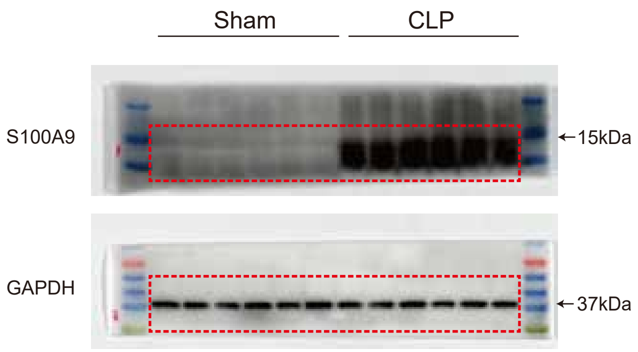

Fig.2F

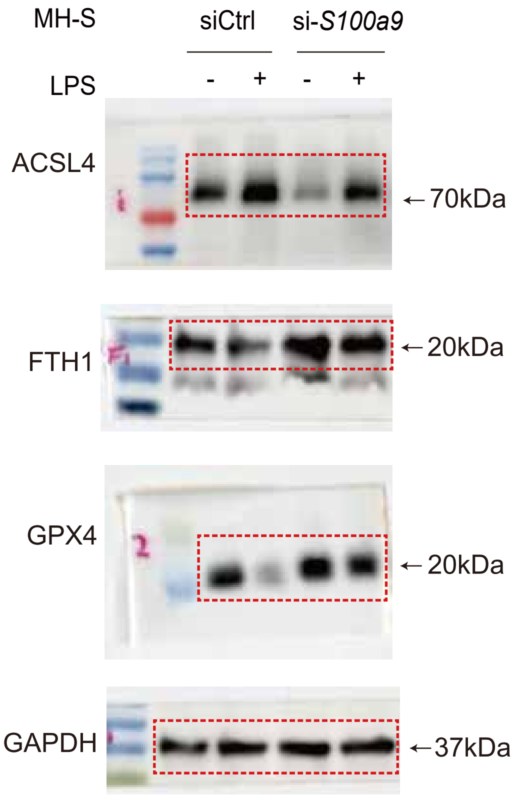

Fig.1B

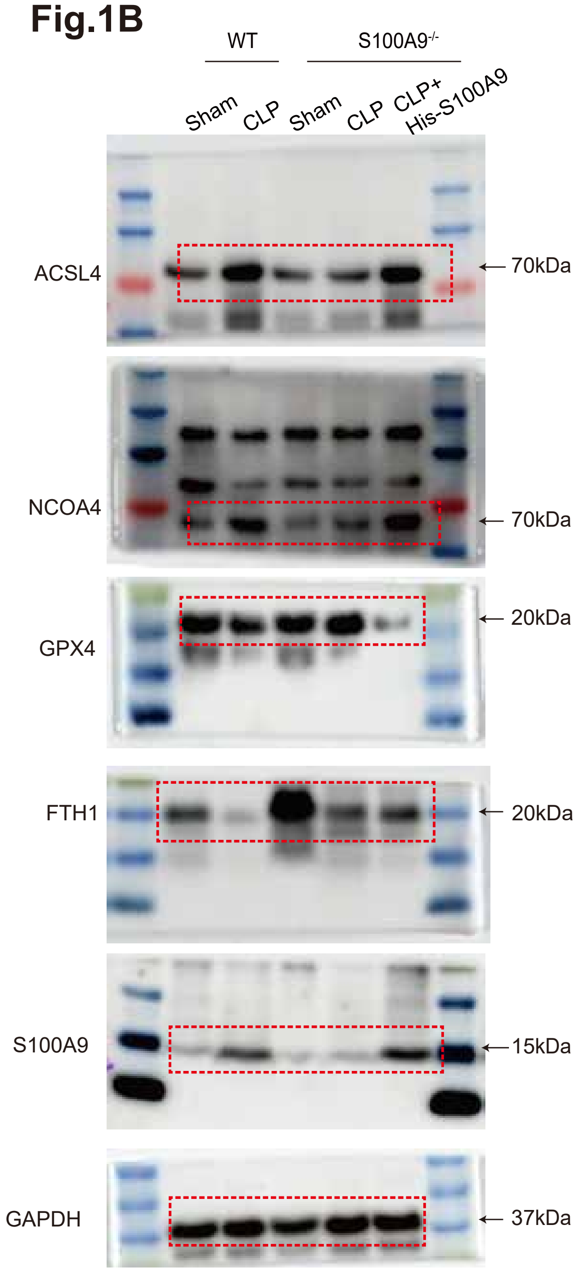

Fig.3A

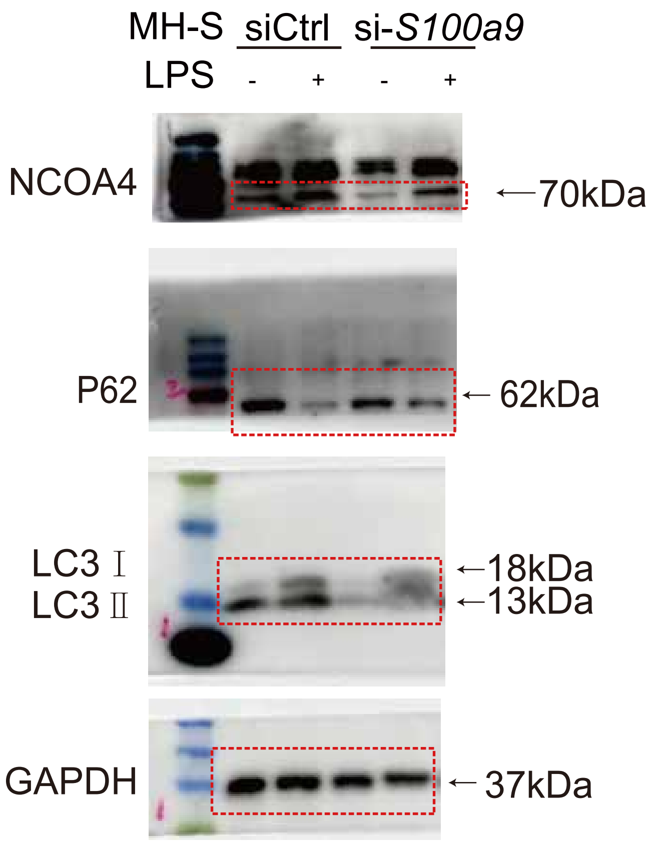

Fig.3B

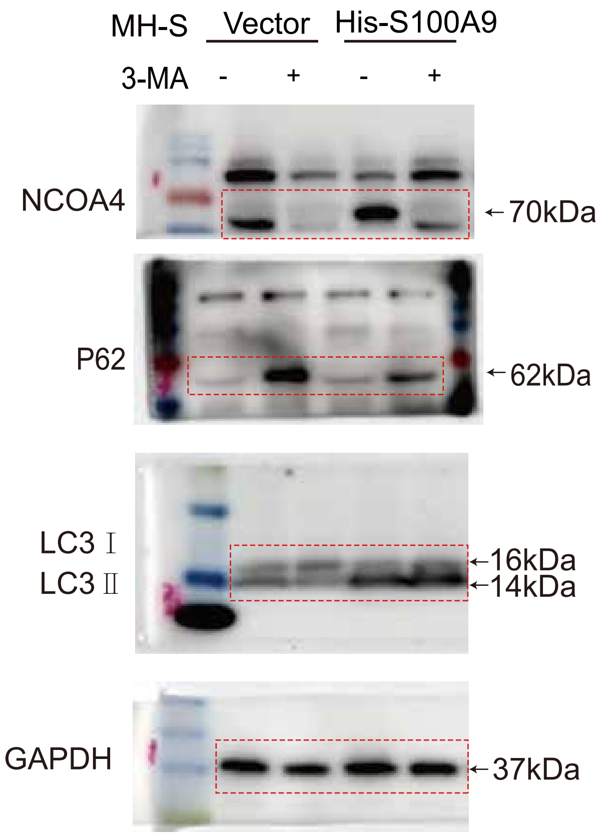

Fig.3G

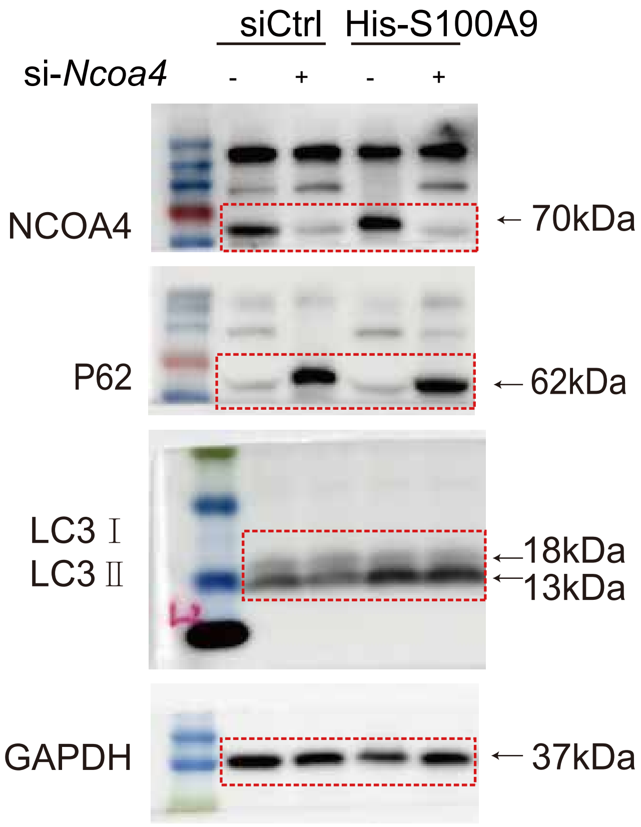

Fig.4A

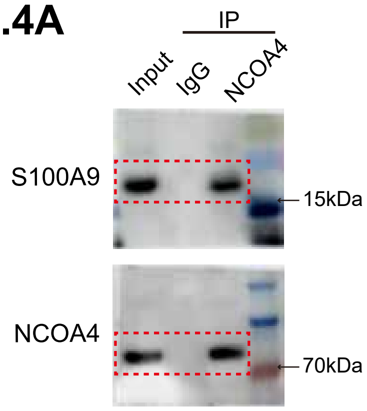

Fig.4B

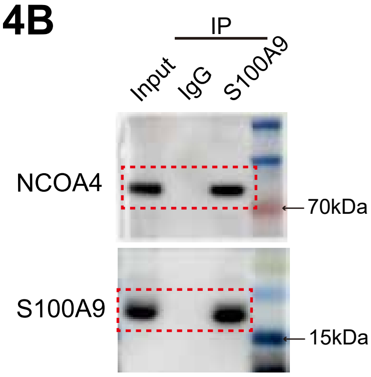

Fig.4C

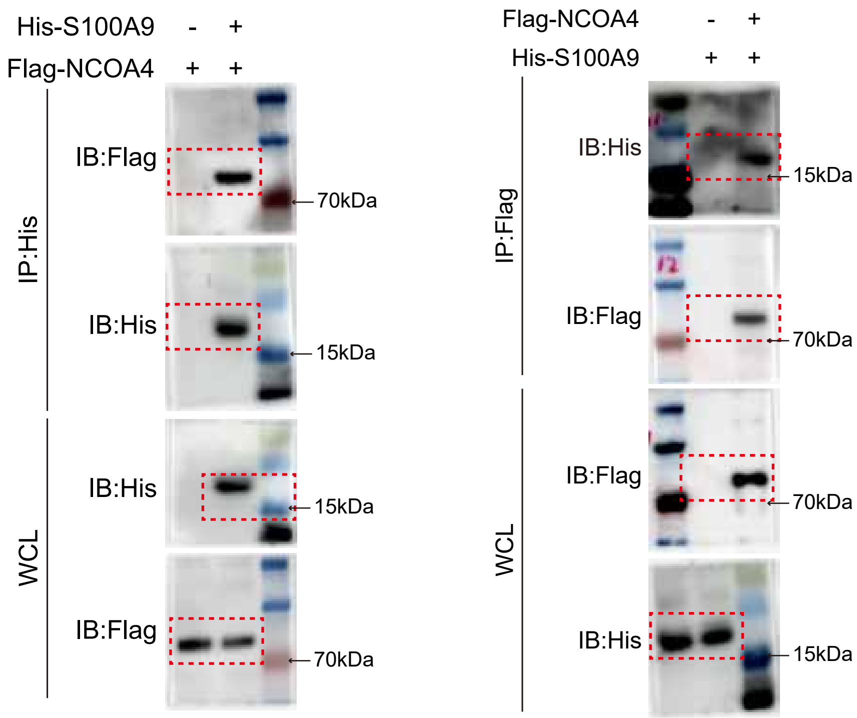

Fig.4F

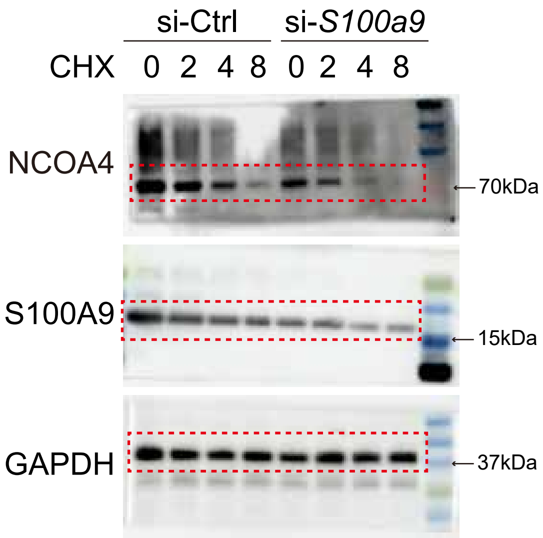

Fig.4G

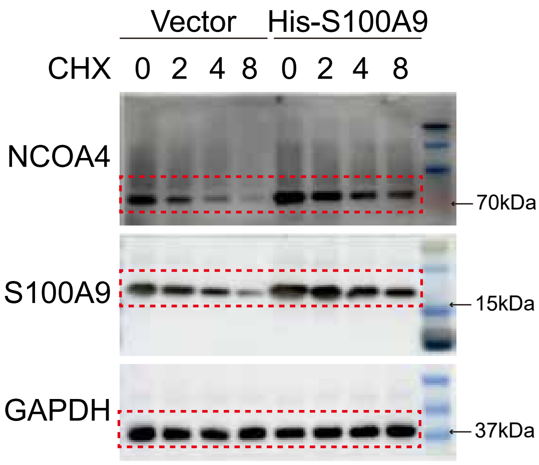

Fig.4H

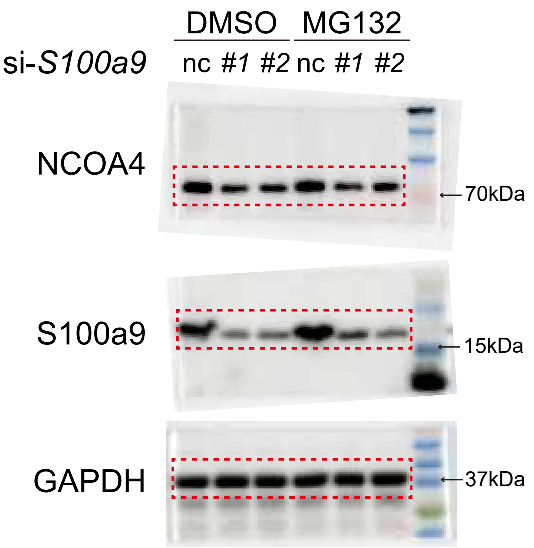

Fig.4I

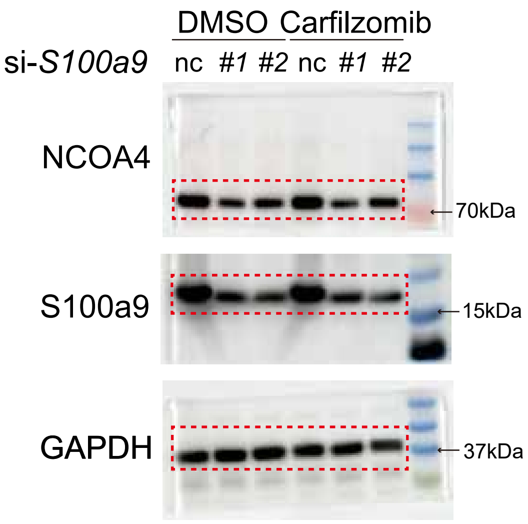

Fig.4J

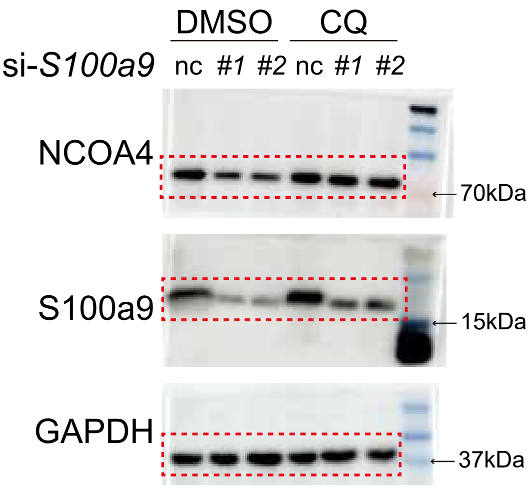

Fig.4K

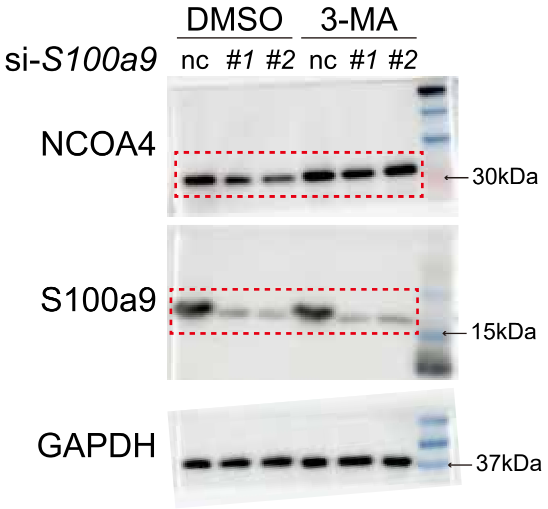

Fig.4M

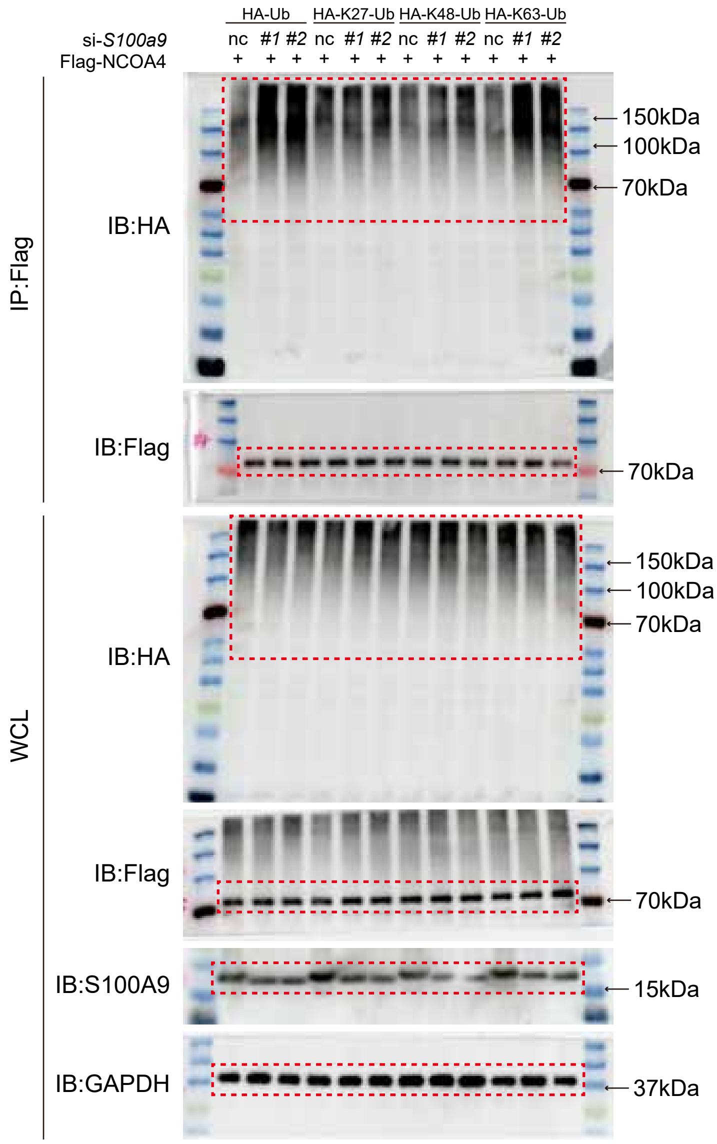

Fig.4N

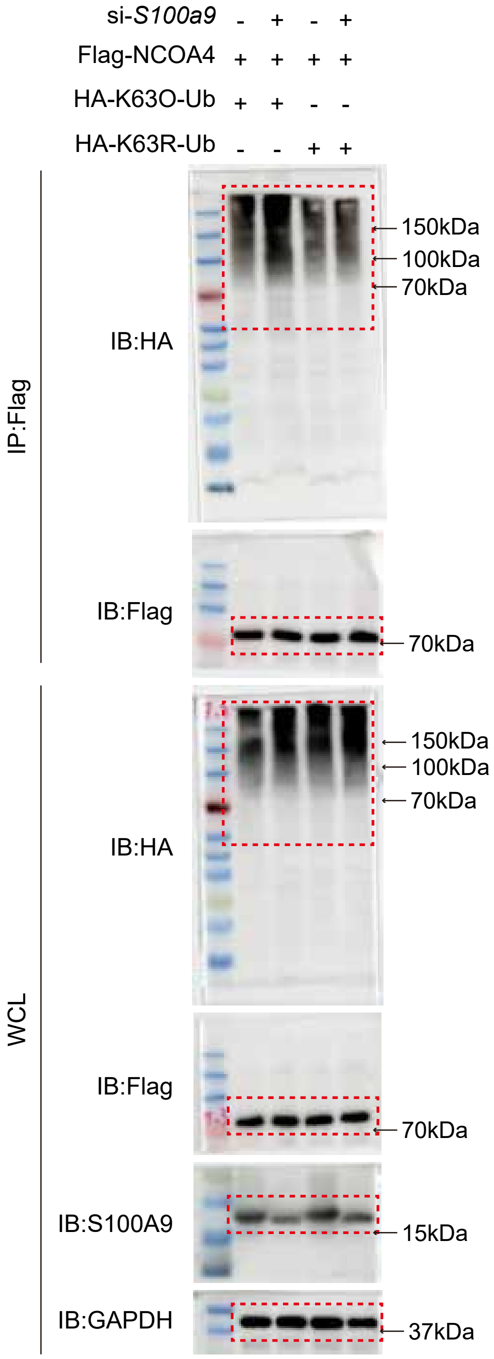

Fig.5B

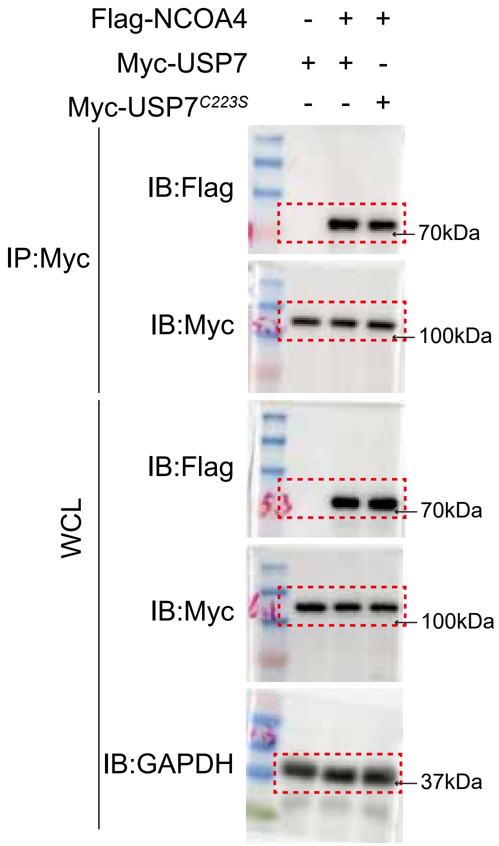

Fig.5C

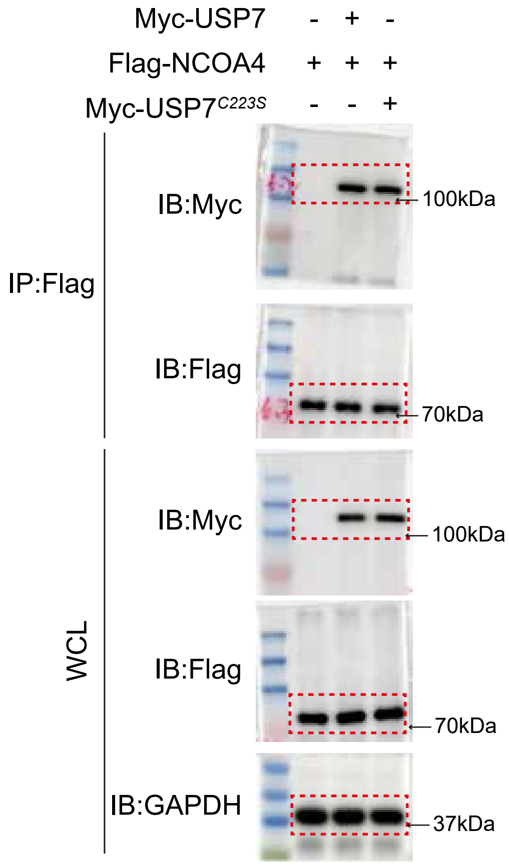

Fig.5E

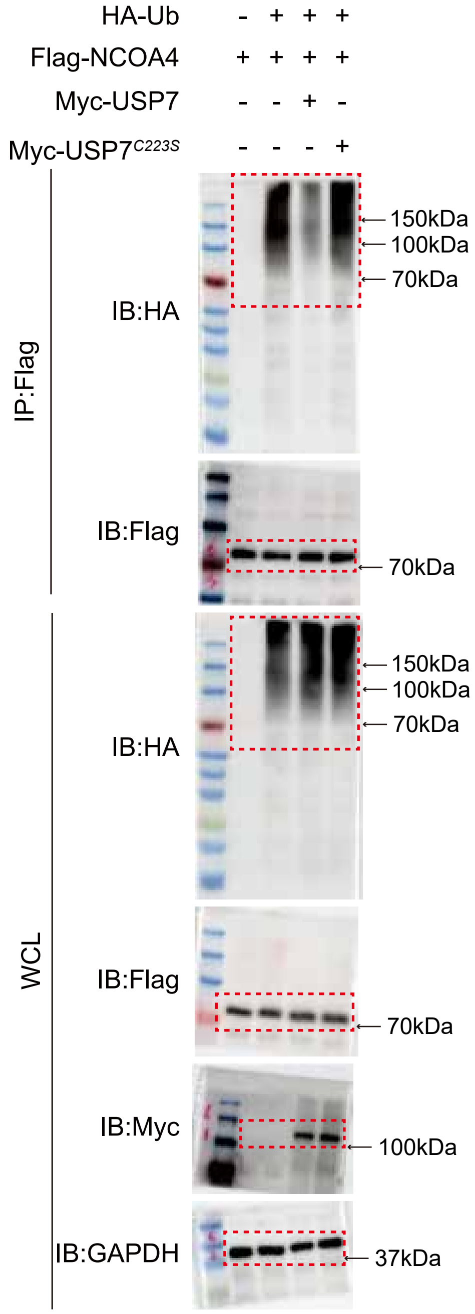

Fig.5D

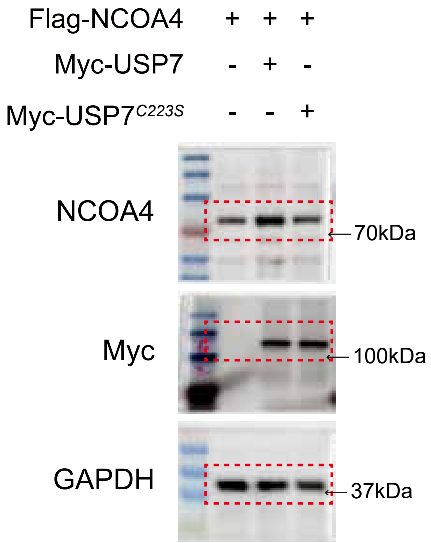

Fig.5H

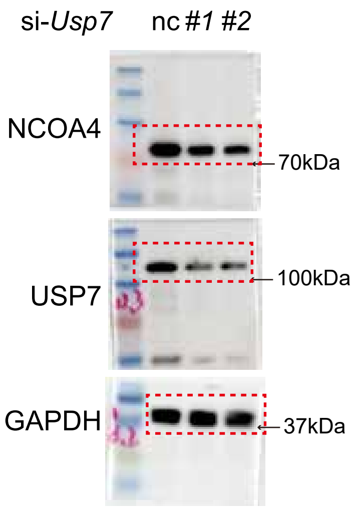

Fig.5K

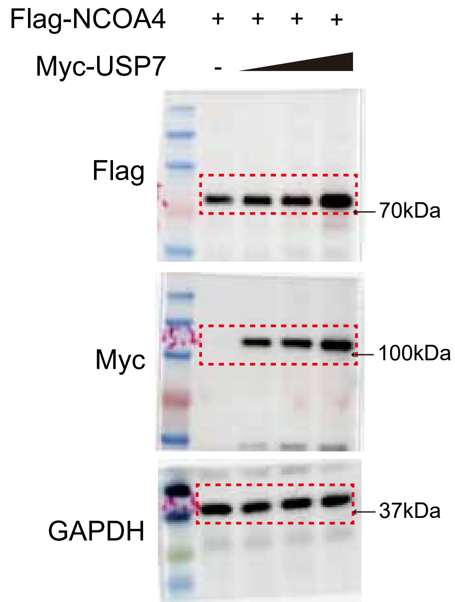

Fig.5N

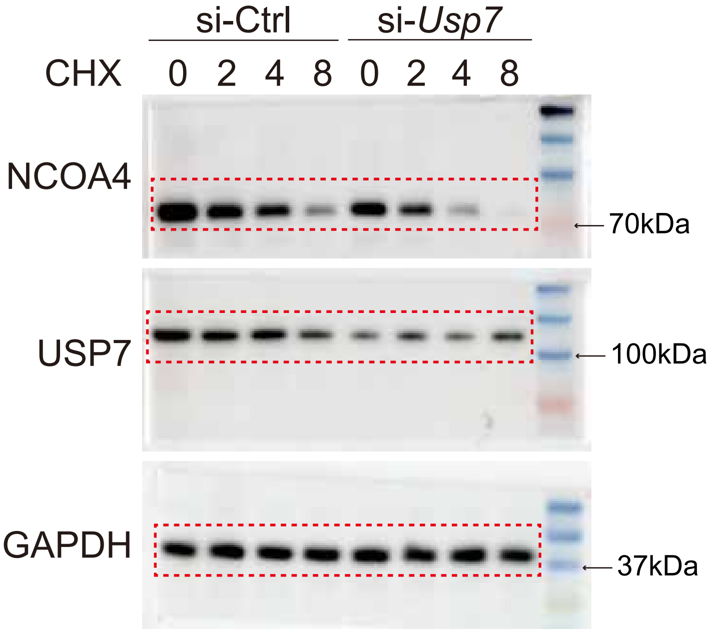

Fig.5P

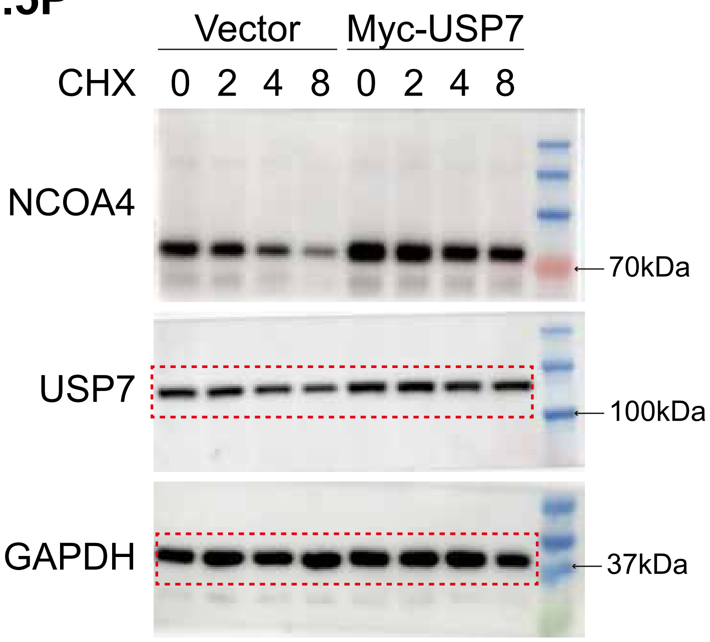

Fig.5R

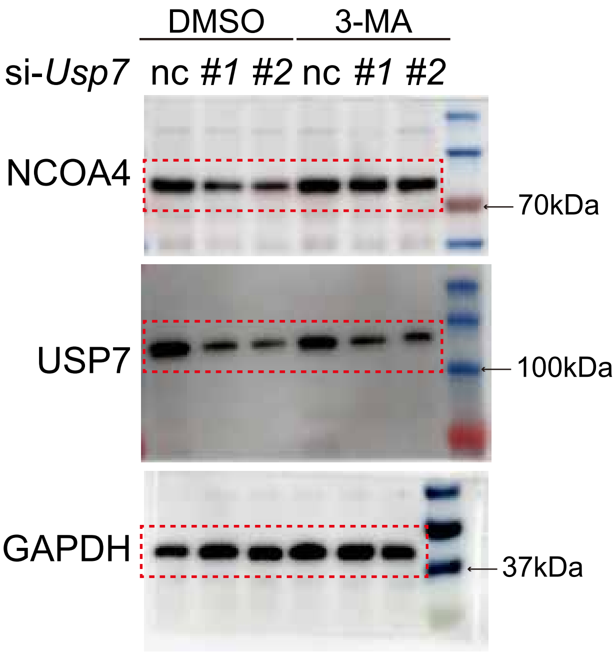

Fig.6A

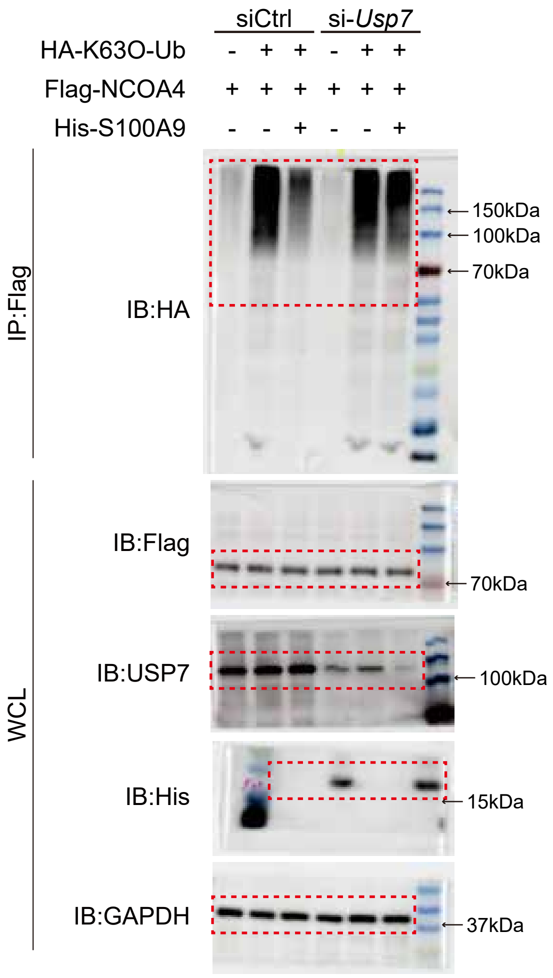

Fig.6B

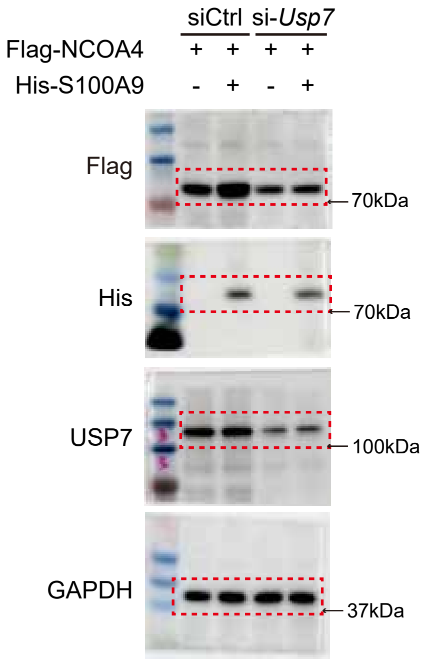

Fig.6C

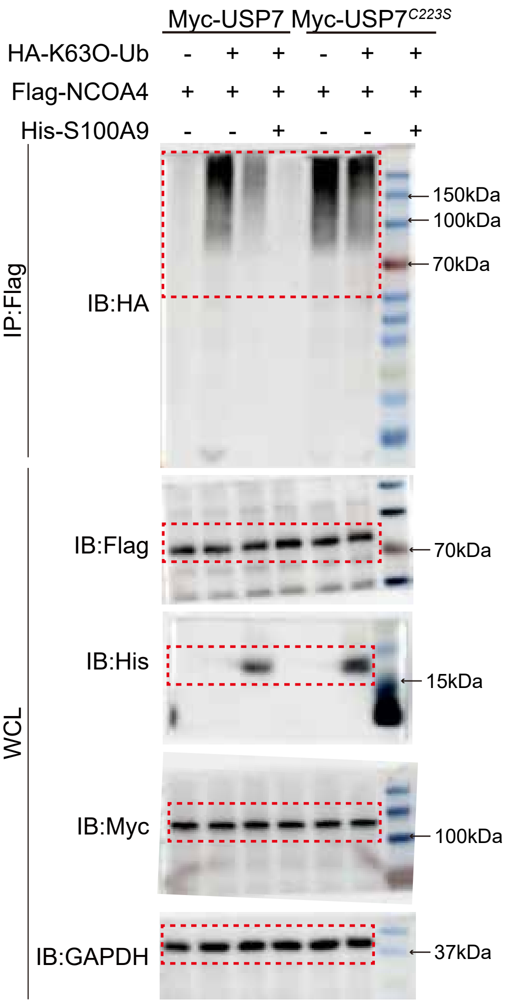

Fig.6D

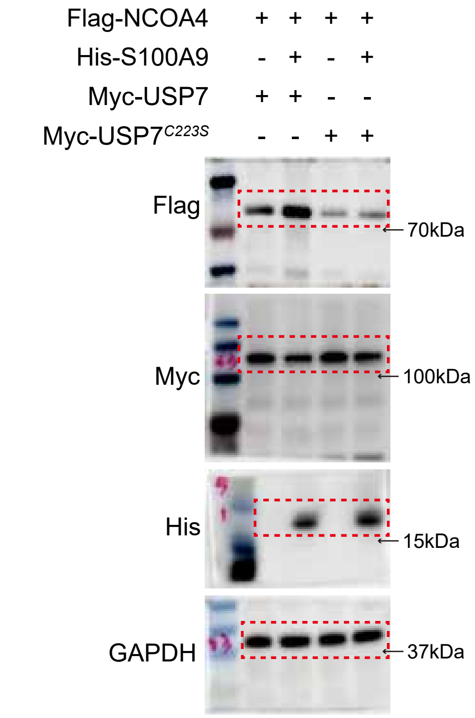

Fig.6E

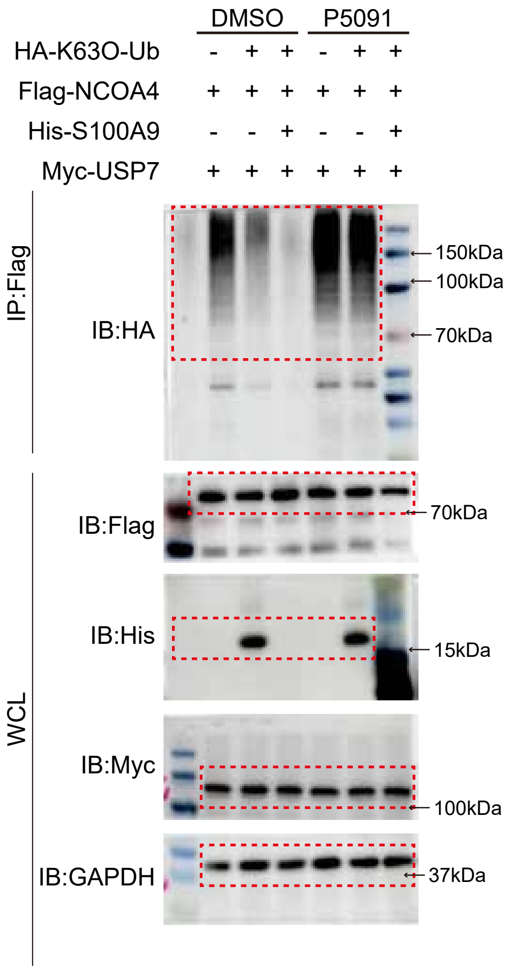

Fig.6F

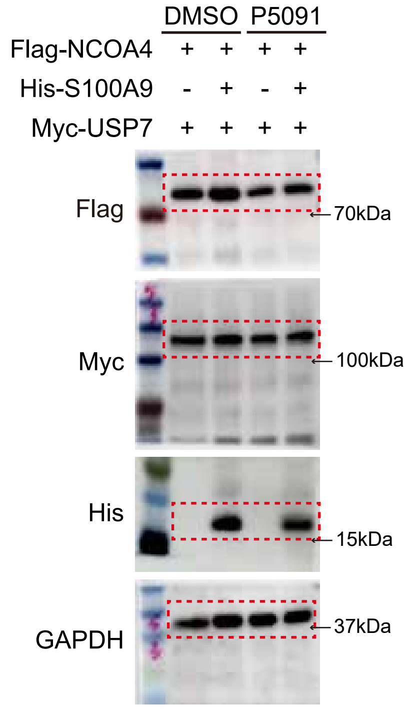

Fig.6H

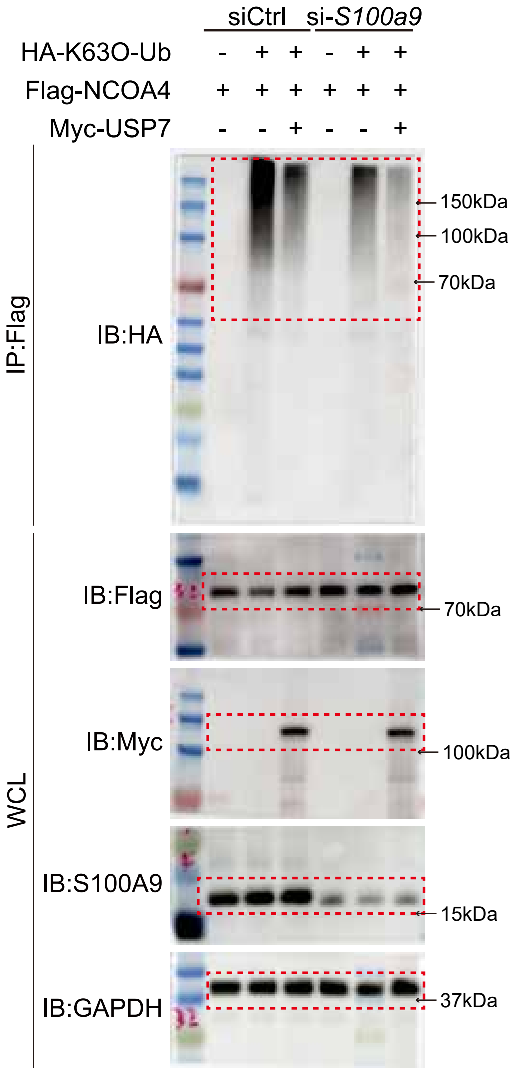

Fig.6I

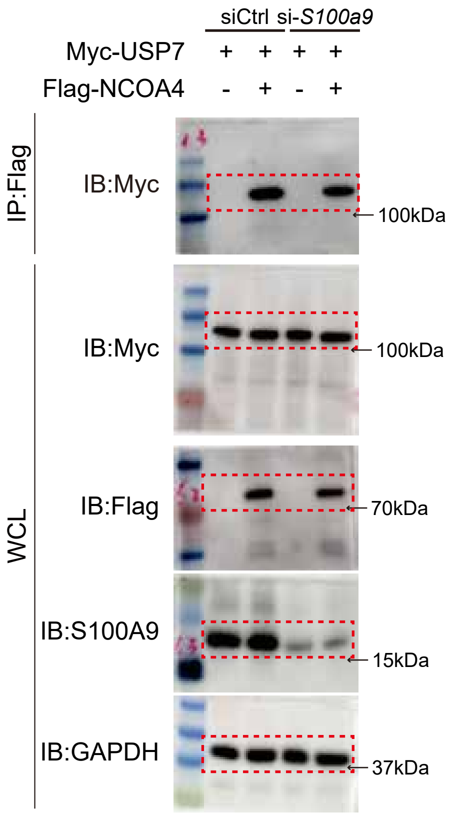

Fig.6G

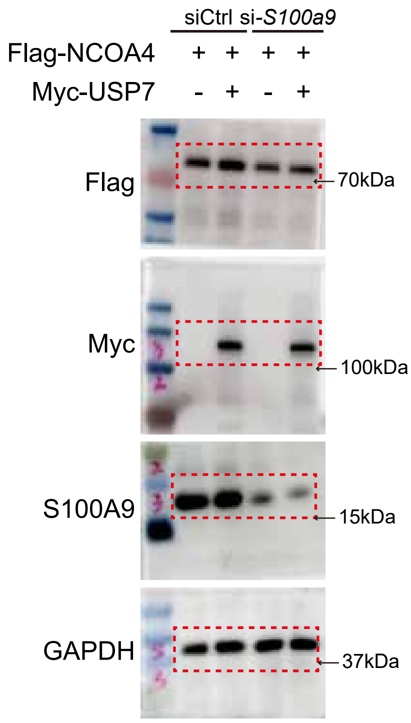

Fig.6J

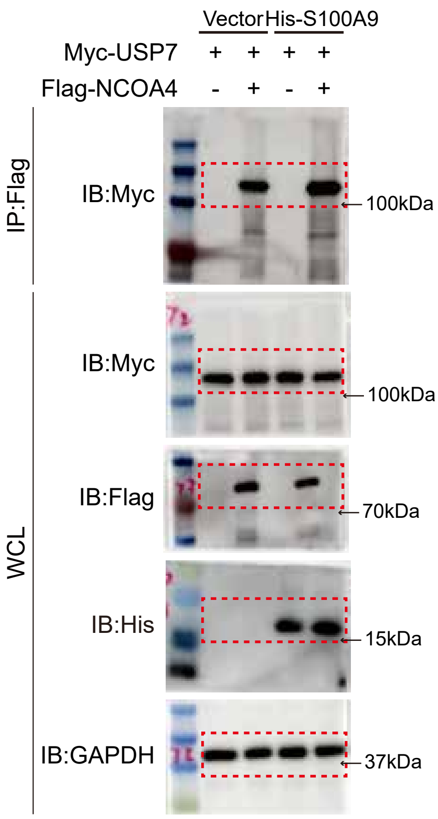

Fig.6K

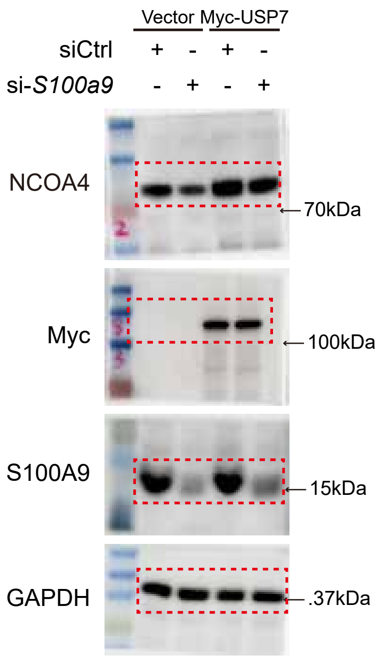

Fig.6L

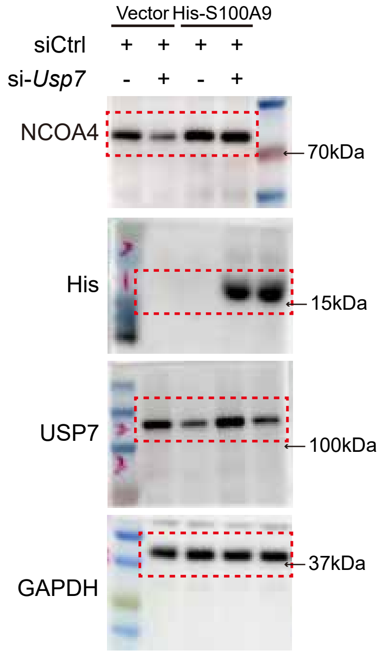

Fig.7C

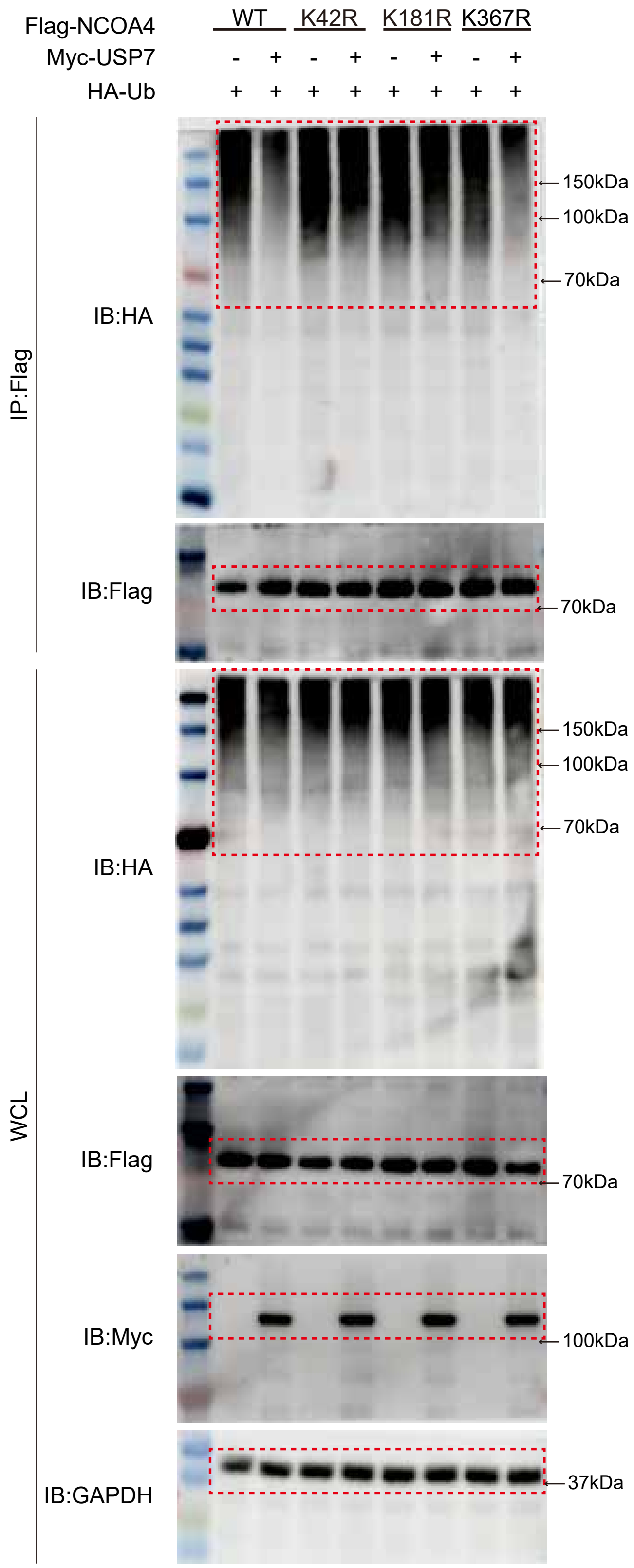

Flag-NCOA4
WT
K42R
K181R
K367R
Myc-USP7
- + - + - + - +
HA-Ub
+ + + + + + + +
IP:Flag
IB:HA
150kDa
100kDa
70kDa
IB:Flag
70kDa
WCL
IB:HA
150kDa
100kDa
70kDa
IB:Flag
70kDa
IB:Myc
100kDa
IB:GAPDH
37kDa

Fig.7D

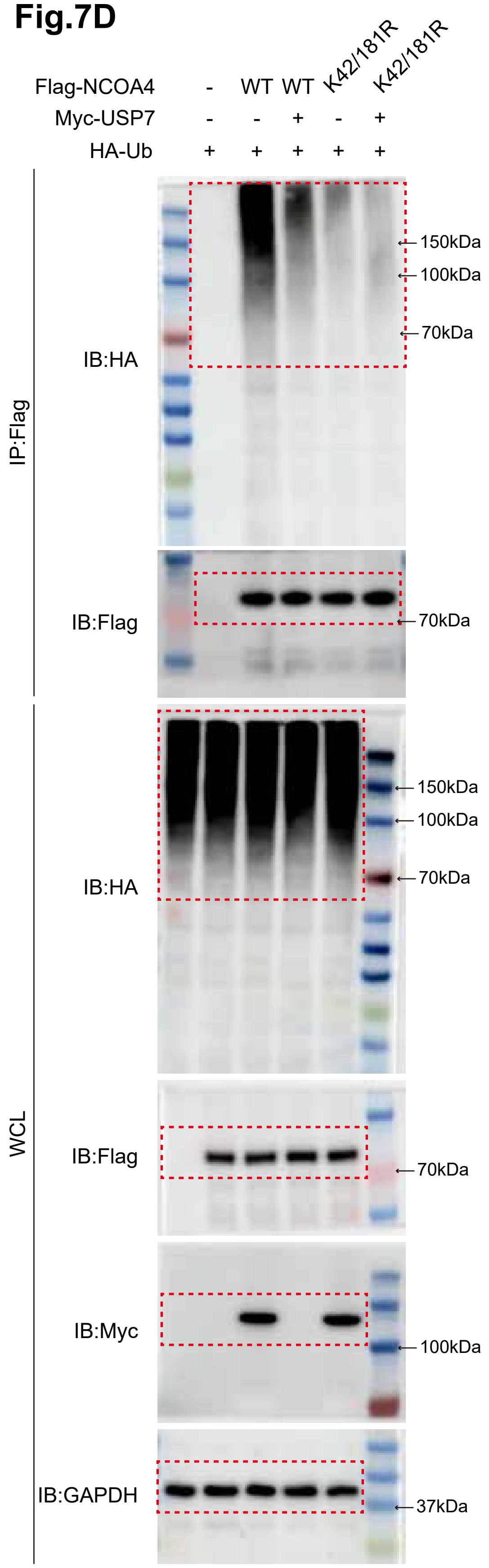

Flag-NCOA4
- WT WT K42/181R K42/181R
Myc-USP7
- - + - +
HA-Ub
+ + + + +
IP:Flag
IB:HA
150kDa
100kDa
70kDa
IB:Flag
70kDa
WCL
IB:HA
150kDa
100kDa
70kDa
IB:Flag
70kDa
IB:Myc
100kDa
IB:GAPDH
37kDa

Fig.7E

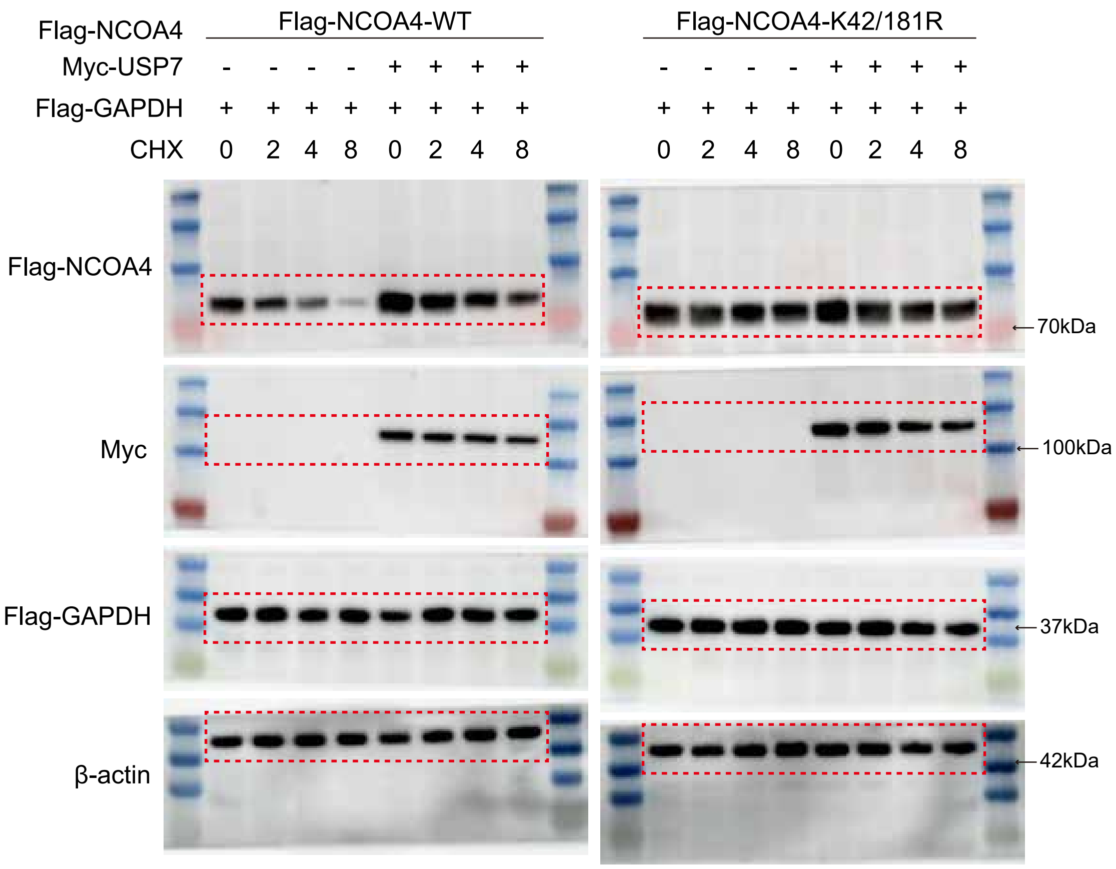

Fig.7F

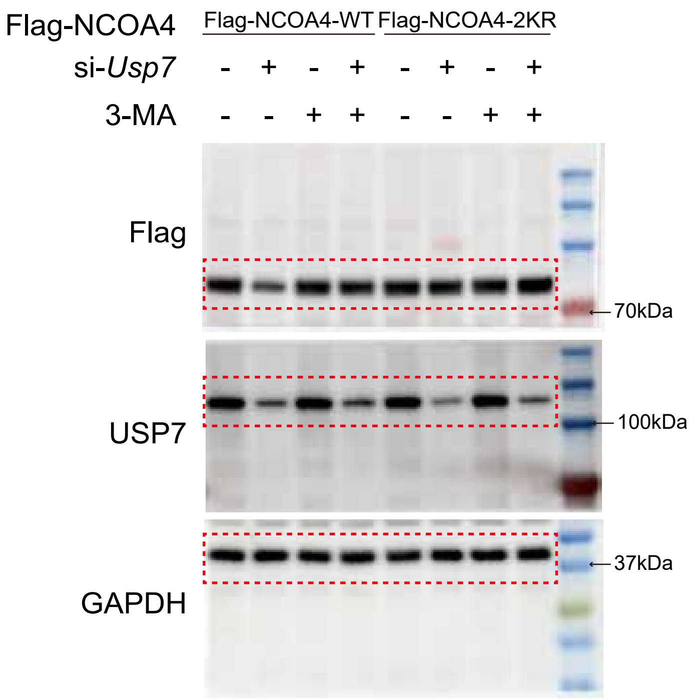

Fig.8L

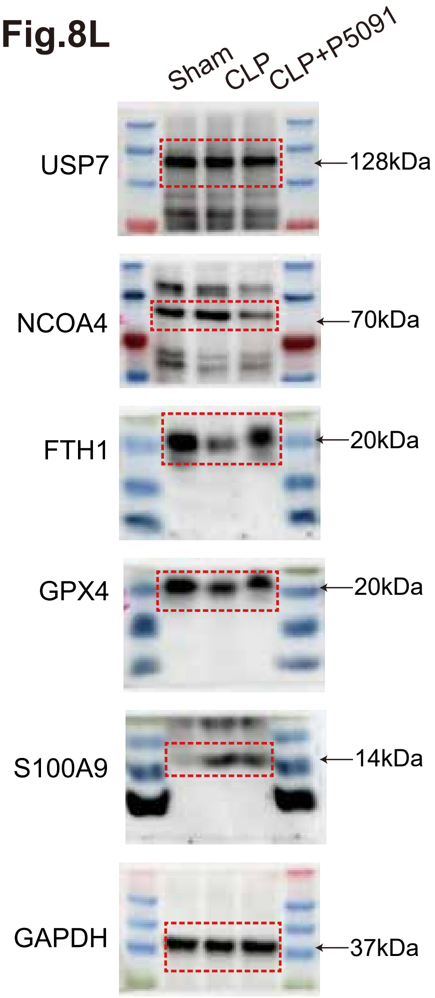

Fig.S2

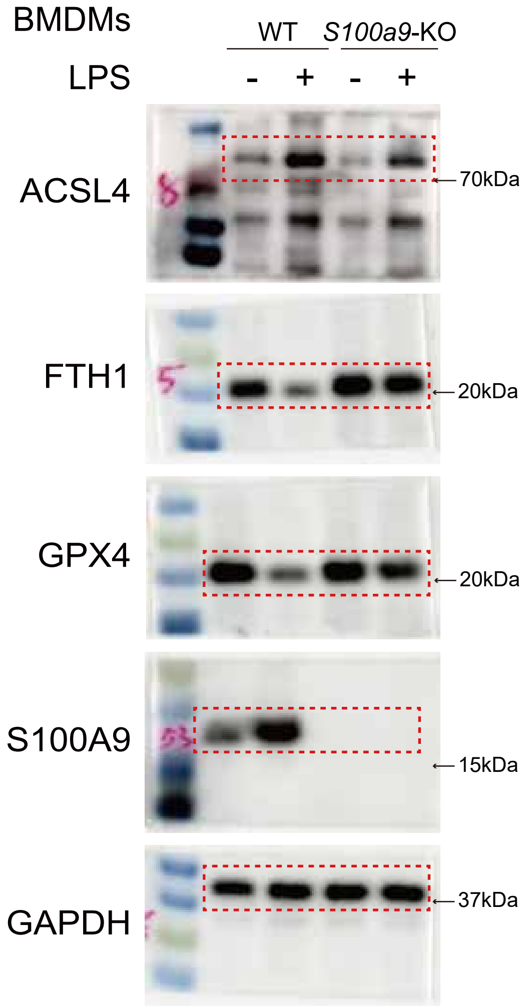

Fig.S4

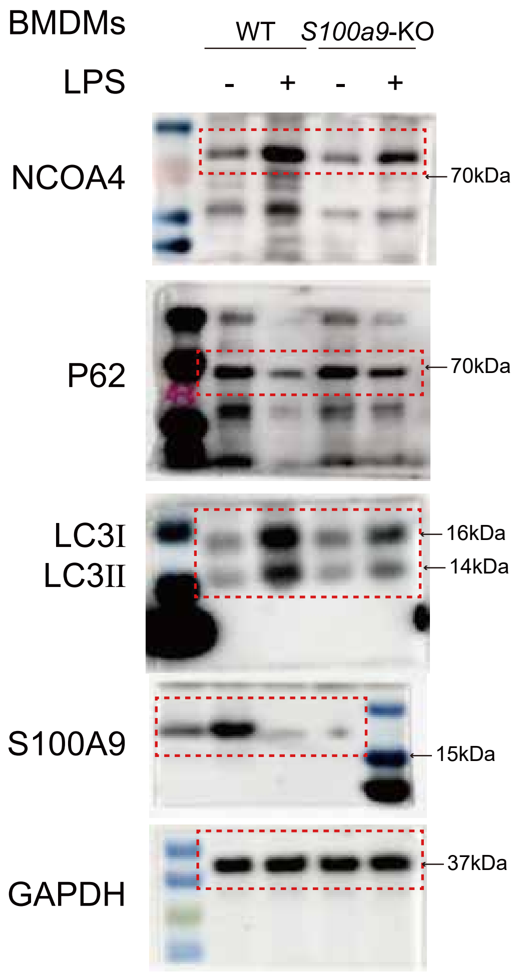

Fig.S5

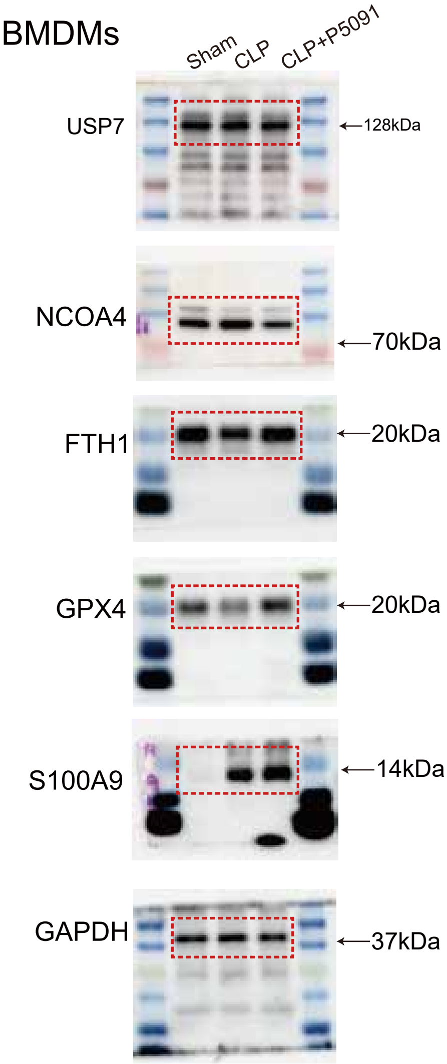

Supplement: Multimedia component 2 [file mmc2.pdf]
